# Overexpression of HMGA1 confers radioresistance by transactivating RAD51 in cholangiocarcinoma

**DOI:** 10.1038/s41420-021-00721-8

**Published:** 2021-10-29

**Authors:** Jianping Song, Donghai Cui, Jing Wang, Junchao Qin, Shourong Wang, Zixiang Wang, Xiangyu Zhai, Huan Ma, Delin Ma, Yanfeng Liu, Bin Jin, Zhaojian Liu

**Affiliations:** 1 Key Laboratory of Experimental Teratology, Ministry of Education, Department of Cell Biology, School of Basic Medical Sciences, Department of Hepatobiliary Surgery, Qilu Hospital, Cheeloo College of Medicine, Shandong University, 107 Wenhua Xi Road, 250012 Jinan, Shandong Province China; 2 Department of Organ Transplantation, Qilu Hospital, Cheeloo College of Medicine, Shandong University, 107 Wenhua Xi Road, 250012 Jinan, Shandong Province China

**Keywords:** Cancer, DNA damage and repair

## Abstract

Cholangiocarcinomas (CCAs) are rare but aggressive tumors of the bile ducts. CCAs are often diagnosed at an advanced stage and respond poorly to current conventional radiotherapy and chemotherapy. High mobility group A1 (HMGA1) is an architectural transcription factor that is overexpressed in multiple malignant tumors. In this study, we showed that the expression of HMGA1 is frequently elevated in CCAs and that the high expression of this gene is associated with a poor prognosis. Functionally, HMGA1 promotes CCA cell proliferation/invasion and xenograft tumor growth. Furthermore, HMGA1 transcriptionally activates RAD51 by binding to its promoter through two HMGA1 response elements. Notably, overexpression of HMGA1 promotes radioresistance whereas its knockdown causes radiosensitivity of CCA cells to X-ray irradiation. Moreover, rescue experiments reveal that inhibition of RAD51 reverses the effect of HMGA1 on radioresistance and proliferation/invasion. These findings suggest that HMGA1 functions as a novel regulator of RAD51 and confers radioresistance in cholangiocarcinoma.

## Introduction

Cholangiocarcinoma (CCA) is the second most common primary hepatic malignancy after hepatocellular carcinoma (HCC) [1]. The incidence and prevalence of CCA have been increasing worldwide in the past several decades. China is one of the regions with the highest incidence of CCA with an incidence >6 per 100,000 habitants per year [2]. CCAs are usually diagnosed at a late stage, and approximately 65% of patients lose the opportunity for surgical treatment [3]. The survival rate of patients with CCA remains poor, with a median overall survival of approximately 6 months [4]. Surgical resection remains the mainstay of potentially curative treatment of early-stage CCA. It is difficult to find a suitable treatment for patients with advanced or recurrent disease. Chemotherapy or radiotherapy does not show satisfactory responses [5]. Therefore, a better understanding of the molecular pathogenesis involved in progression and drug resistance may unveil diagnostic markers and identify effective therapeutic strategies.

High mobility group A1 (HMGA1) is a nuclear protein that preferentially binds to the minor groove of many AT-rich promoters and enhancer DNA regulatory elements [6]. The expression level of HMGA1 is low or absent in adult normal tissues, whereas it is highly expressed during embryonic development [7]. HMGA1 is frequently overexpressed in various human malignancies, including breast cancer and CCA [8, 9]. HGMA1 is involved in neoplastic transformation, metastasis, and treatment resistance in various malignancies [6, 10, 11]. HMGA1 could directly bind to promoters and activate the expression of several genes. HMGA1 binds to AT-rich regions and activates INSR (insulin receptor), COX-2 and CCNB2 expression [12–14]. HMGA1 could also modulate autophagy by binding to the ULK1 promoter region and negatively regulating its transcription [15].

Emerging evidence shows that HMGA1 plays a role in DNA repair. HMGA1 sensitizes breast cancer cells to cisplatin by downregulating the expression of BRCA1 [4]. HMGA1 inhibits global genomic nucleotide excision repair (NER) induced by UV radiation [16]. In contrast, HMGA1 overexpression promotes chemoresistance to cetuximab and 5-fluorouracil in colon and thyroid cancer cells [17]. HMGA1 enhances the recovery from double-strand breaks (DSBs) through nonhomologous end-joining (NHEJ) DNA repair [18]. Thus, although the roles of HMGA1 in DNA repair are controversial, its expression and functional role in cholangiocarcinoma remain unclear.

In the present study, we observed that HMGA1 was commonly upregulated in cholangiocarcinoma and was associated with a poor prognosis. We further demonstrated that HMGA1 transcriptionally activates RAD51 by binding to its promoter in CCA. Importantly, HMGA1 inactivation sensitizes CCA cells to X-ray irradiation, and overexpression of RAD51 attenuates radiosensitivity. Our data suggest that HMGA1-mediated RAD51 upregulation contributes to radioresistance in CCA.

## Results

### High HMGA1 expression is associated with a poor prognosis in patients with CCA

**Fig. 1:**
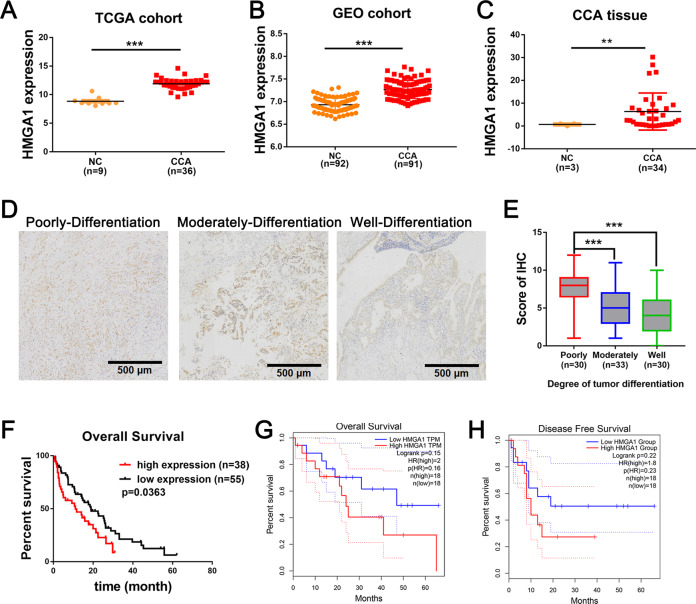
HMGA1 is frequently upregulated in CAAs and is correlated with a poor prognosis. **A**–**C** Expression analysis of HMGA1 in TCGA CAA, GSE76297 and Qilu hospital cohorts. **D** Representative images of immunohistochemical (IHC) staining of HMGA1 in our tissue microarray (containing 93 samples of CCA tissues collected from Qilu Hospital, Shandong University). **E** Statistical analysis of HMGA1 expression from IHC staining of our tissue microarray. High and low expression indicate the final score of each sample determined by two pathologists based on the intensity and extent of staining across the tissue microarray section. CCA patients were dichotomized into low (Score < 7) and high (Score ≧ 7) HMGA1 protein expression groups based on the IHC staining score. The high and low expression groups were separated based on the best cutoff. **F** Kaplan–Meier analysis of the correlation between HMGA1 expression and clinical prognosis based on data from our tissue microarray. **G**, **H** Kaplan–Meier analysis of the correlation between HMGA1 expression and the clinical prognosis (http://gepia.cancer-pku.cn/index.html). *P*-values were obtained by Student’s *t* test (**A**–**C**, **H**, **I**) or log-rank test (**D**–**F**). *P < 0.05, **P < 0.01, ***P < 0.001.

To investigate the expression and clinical implications of HMGA1 in CCAs, we first analyzed HMGA1 mRNA expression using TCGA-CCA data and found that the HMGA1 expression level was higher in CCAs (*n* = 36) than in paracancerous tissues (*n* = 9) (Fig. 1A). Further analysis in an independent cohort (GSE76297) confirmed overexpression of HMGA1 in CCAs (*n* = 91) compared to normal tissues (*n* = 92) (Fig. 1B). We subsequently validated HMGA1 expression in our cohort by qPCR, and the results showed that HMGA1 was frequently upregulated in CCAs (*n* = 34) compared to normal tissues (*n* = 3), as shown in Fig. 1C. Pan-cancer analysis revealed that HMGA1 was upregulated in various cancer types including CCA (Fig. S1A). To evaluate the clinical significance of HMGA1 in CCAs, we performed immunohistochemistry in CCAs (*n* = 93) and analyzed the clinicopathologic parameters. As a result, the expression of HMGA1 was correlated with the degree of tumor differentiation, and CCAs with poor differentiation exhibited higher expression of HMGA1 (Fig. 1D, E). We further assessed the prognostic value, and the results of Kaplan–Meier survival analysis indicated that high HMGA1 expression was correlated with an unfavorable prognosis in CCA patients (Fig. 1F). The prognostic value of HMGA1 in CCAs was further verified using the TCGA-CCA cohort, and a high level of HMGA1 was correlated with poor overall survival and disease-free survival (Fig. 1G, H). Moreover, high HMGA1 expression was significantly correlated with lymph node metastasis and CA199 in CCAs (Table S1-2). These findings strongly indicate that overexpression of HMGA1 is associated with a poor prognosis in patients with CCA.

### HMGA1 promotes CCA cell proliferation/invasion and xenograft tumor growth

**Fig. 2:**
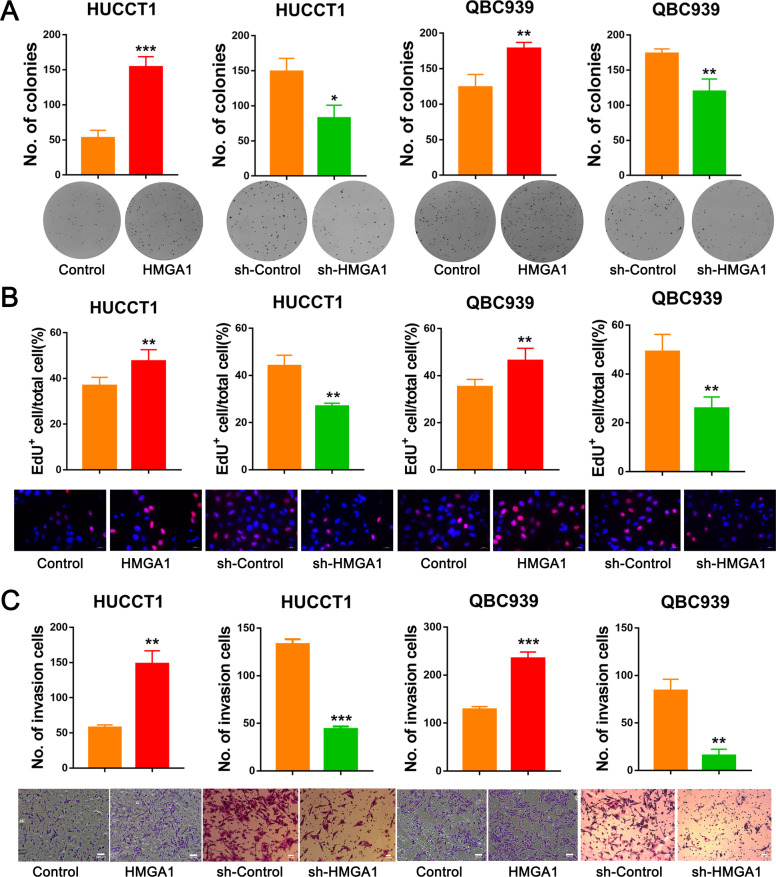
HMGA1 promotes the proliferation and invasion of CCA cells. **A**, **B** The effect of HMGA1 on the proliferation of CCA cells was examined by clonogenic (**A**) and EdU (**B**) assays of CCA cells with HMGA1 overexpression or knockdown (*n* = 3 biologically independent samples). **C** Matrigel invasion data showing that overexpression of HMGA1 significantly enhanced the invasion capacity, whereas knockdown of HMGA1 decreased the invasion potential of CCA cells (*n* = 3 biologically independent samples). The *P*-value was obtained by Student’s *t* test. The results represent the mean ± S.D. of three independent experiments. *P < 0.05, **P < 0.01, ***P < 0.001.

To explore the functional role of HMGA1 in CCA, we established stable cell lines with HMGA1 overexpression or knockdown. We then conducted a clonogenic assay to evaluate the effect of HMGA1 on the colony formation of CCA cells. Overexpression of HMGA1-enhanced clonogenic capacity in HUCCT1, QBC939 and HCCC-9810 cells, while knockdown of HMGA1 significantly reduced their colony-forming efficiency (Fig. 2A and Fig. S1B). We then conducted an EdU assay and found that HMGA1 overexpression significantly increased the number of EdU-positive cells, while knockdown of HMGA1 decreased the ratio of EdU-positive cells compared with the control group (Fig. 2B; Fig. S1C, D). As higher expression of HMGA1 in CCA patients was associated with increased lymph node metastasis, we speculated that HMGA1 may promote the invasiveness of CCA cells. We performed a transwell assay and found that HMGA1 overexpression significantly enhanced the invasion potential, while knockdown of HMGA1 led to reduced invasion compared with control cells (Fig. 2C and Fig. S1E).

**Fig. 3:**
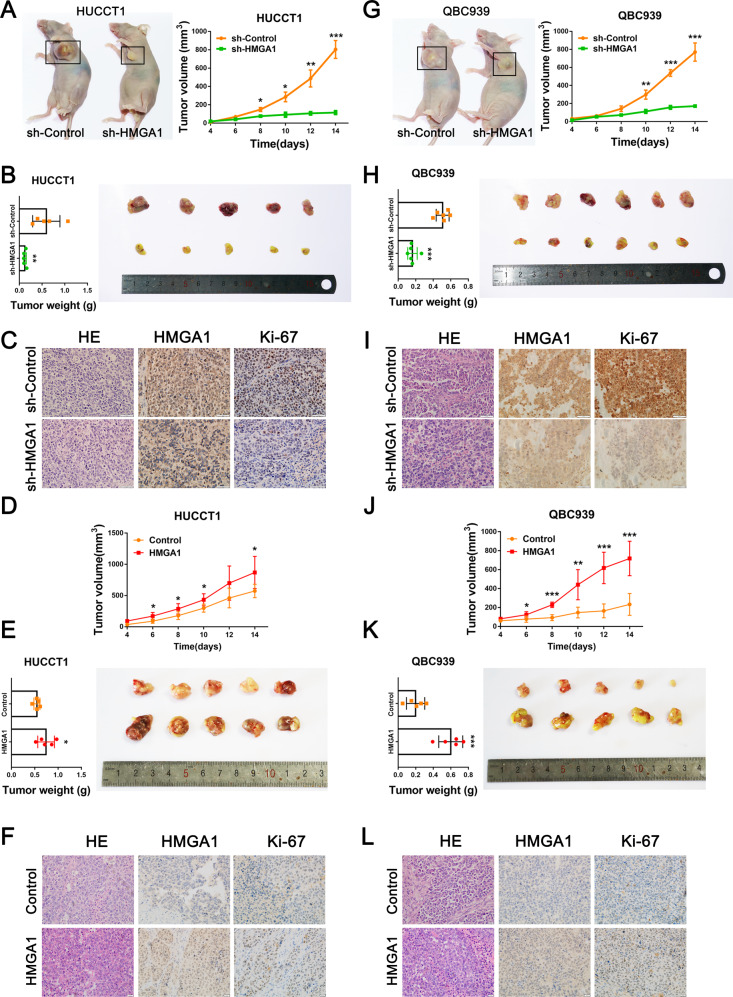
HMGA1 promotes tumorigenesis and progression of CCA in nude mouse xenografts. **A**, **B** Images of xenograft tumors, tumor mass, and tumor volume in mice subcutaneously injected with HUCCT1 cells with HMGA1 knockdown compared to the corresponding control (*n* = 5 mice per group). **C** Representative images of HE staining and IHC staining for HMGA1 and Ki-67 in HUCCT1 cell xenograft tumors. **D**, **E** Images of xenograft tumors, tumor mass, and tumor volume in mice subcutaneously injected with HUCCT1 cells with HMGA1 overexpression compared to the corresponding control (*n* = 5 mice per group). **F** Representative images of HE staining and IHC staining for HMGA1 and Ki-67 in HUCCT1 cell xenograft tumors. **G**, **H** Images of xenograft tumors, tumor mass, and tumor volume in mice subcutaneously injected with QBC939 cells with HMGA1 knockdown compared to the corresponding control (*n* = 6 mice per group). **I** Representative images of HE staining and IHC staining for HMGA1 and Ki-67 in QBC939 cell xenograft tumors. **J**, **K** Images of xenograft tumors, tumor mass, and tumor volume in mice subcutaneously injected with QBC939 cells with HMGA1 overexpression compared to the corresponding control (*n* = 5 mice per group). **L** Representative images of HE staining and IHC staining for HMGA1 and Ki-67 in QBC939 cell xenograft tumors. The *P*-value was obtained by Student’s *t* test. The results represent the mean ± S.D. *P < 0.05, **P < 0.01, ***P < 0.001.

We next assessed the potential role of HMGA1 in tumor growth by establishing a subcutaneous xenograft model using HUCCT1 (*n* = 5) and QBC939 cells (*n* = 6) with HMGA1 knockdown or overexpression. As a result, knockdown of HMGA1 remarkably reduced the tumor volume and weight in both cell line xenografts (Fig. 3A, B, G, H). Consistent with this, the number of Ki-67-positive cells was significantly decreased in tumors with HMGA1 depletion (Fig. 3C, I). Furthermore, HUCCT1 and QBC939 cells with HMGA1 overexpression were also subcutaneously injected into nude mice (*n* = 5) and forced expression of HMGA1 induced a significant increase in tumor mass and volume in xenografts of both cell lines (Fig. 3D, E, J, K). As expected, Ki-67-positive cells were increased in tumors with HMGA1 overexpression (Fig. 3F, L). These results strongly suggest that HMGA1 promotes CCA cell proliferation/invasion and xenograft tumor growth.

### HMGA1 knockdown altered DNA repair genes, including RAD51

**Fig. 4:**
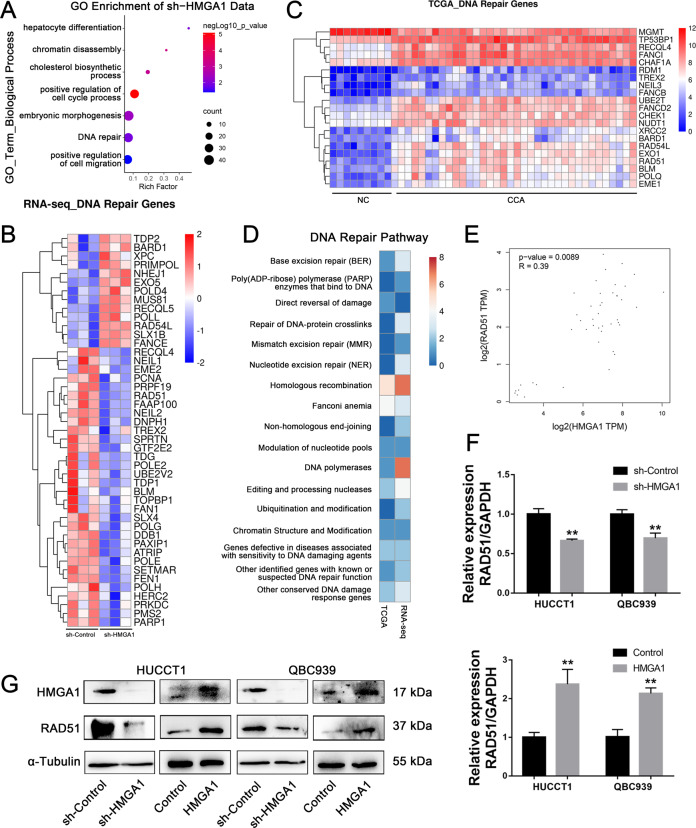
HMGA1 positively regulates RAD51 expression in CCA cells. **A** Gene Ontology (GO) analysis of differentially expressed genes (2473 up- and 2058 downregulated) in RNA-seq data of HUCCT1 cells with HMGA1 knockdown. **B**, **C** Forty-five and 21 differentially expressed DNA repair genes were filtered from RNA-seq data and TCGA data, respectively. **D** The homologous recombination pathway was enriched in both RNA-seq and TCGA data. **E** Correlation analysis of HMGA1 and RAD51 expression in TCGA-CCA samples (*R* = 0.39, *P* = 0.0089). **F** Validation of the expression of RAD51 in HUCCT1 and QBC939 cell lines with HMGA1 overexpression and knockdown by qPCR. **G** The protein levels of RAD51 and HMGA1 were measured by western blot in HUCCT1 and QBC939 cells with HMGA1 overexpression or knockdown. The *P*-value was obtained by Student’s *t* test. The results represent the mean ± S.D. of three independent experiments. *P < 0.05, **P < 0.01, ***P < 0.001.

To explore HMGA1-regulated targets and signaling pathways involved in the regulation of CCA progression, we performed RNA-seq in HUCCT1 cells with HMGA1 knockdown compared to control cells. Gene Ontology enrichment analysis of 4531 differentially expressed genes (2473 up- and 2058 downregulated) revealed that biological processes, including the cell cycle and DNA repair, were significantly enriched (Fig. 4A). We focused our attention on the DNA repair pathway, and hierarchical clustering analysis showed that 45 DNA repair genes were altered in HUCCT1 cells with HMGA1 knockdown (Fig. 4B). CDC25A, which was a known HGMA1 target [19], was also significantly downregulated (Fig. S2A). Additionally, we analyzed DNA repair genes using TCGA data and found that 21 DNA repair genes were significantly overexpressed in CCAs (Fig. 4C). Deep analysis revealed that the homologous recombination repair pathway was highly enriched in both TCGA-CCA and RNA-seq data, including RAD51, RAD54 L and BRAD1 (Fig. 4D and Fig. S2B). Notably, RAD51, which is the key enzyme for homologous recombination, was correlated with HMGA1 in TCGA-CCA (Fig. 4E). We next validated RAD51 expression in CCA cells with HMGA1 overexpression or knockdown. We found that both the mRNA and protein levels of RAD51 were downregulated upon HMGA1 depletion, while they were upregulated after forced expression of HMGA1 in CCA cells (Fig. 4F, G). Similarly, RAD51 was downregulated in breast cancer cell line MDA-MB-231 upon HMGA1 depletion (GSE35525) (Fig. S2C, D). These findings indicate that HMGA1 positively regulates the expression of RAD51 in CCA.

### HMGA1 transcriptionally activates RAD51 by binding to its promoter

**Fig. 5:**
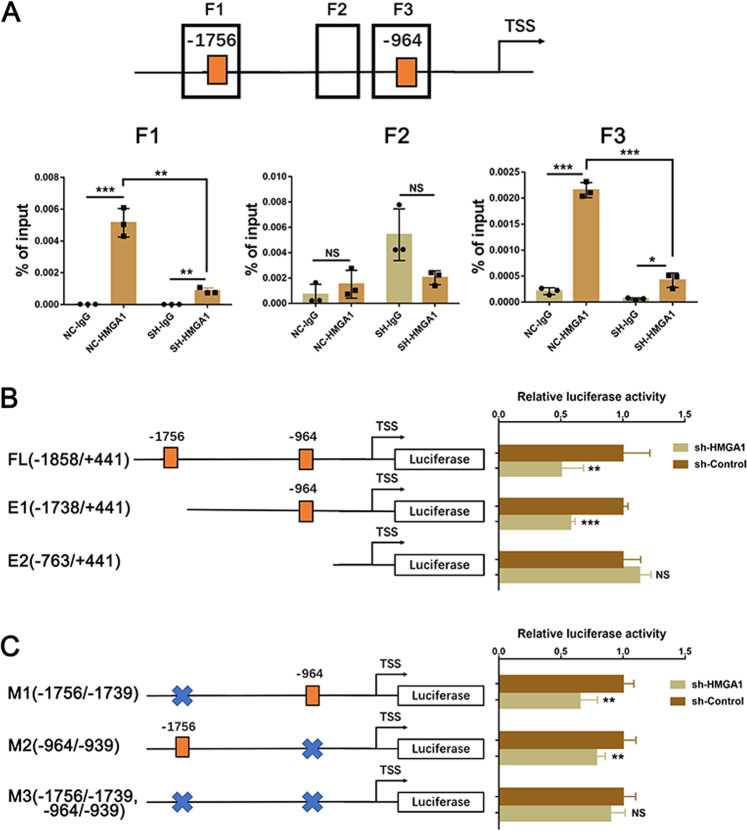
HMGA1 directly binds to the RAD51 promoter and induces its transcription. **A** qPCR analysis of ChIP samples in HUCCT1 cells with or without HMGA1 knockdown using the HMGA1 antibody or IgG. Fragment 1 (F1) and Fragment 3 (F3) were predicted targets of HMGA1, and Fragment 2 (F2) was the negative control. The *P*-value was obtained by Student’s *t* test. The results represent the mean ± S.D. of three independent experiments. **p < 0.01, ***p < 0.001. **B** Luciferase activity was measured in HEK293T cells transfected with HMGA1 knockdown or control plasmids in combination with pGL4 plasmids containing the full long (FL) or truncated (E1 and E2) promoter region (*n* = 5 biologically independent samples). **C** Luciferase activity was measured in HEK293T cells transfected with HMGA1 knockdown or control plasmids in combination with pGL4 plasmids containing the mutation of potential HMGA1 binding sites promoter region (−1756/−1739 M1; −964/−939 M2; double mutant: M3). Data are presented as means ± S.D. *p < 0.05, **p < 0.01, ***p < 0.001, and ****p < 0.0001.

To determine whether HMGA1 regulates RAD51 expression via transcriptional activation, we analyzed the RAD51 promoter region using CONSITE (http://consite.genereg.net/) and found two putative binding sites of HMGA1. We next conducted ChIP-PCR assays with anti-HMGA1 antibody. As expected, HMGA1 was significantly enriched at two putative binding sites in the RAD51 promoter region, and the enrichment levels were reduced upon HMGA1 depletion (Fig. 5A). CDC25A promoter region was included as positive control (Fig. S2E). Furthermore, PGL4.26 plasmids carrying the RAD51 promoter region with the full-length, truncated and mutant fragments were constructed. A luciferase assay was then performed, and the results revealed that HMGA1 knockdown reduced the luciferase activity of full-length and E1 fragments with putative binding sites, whereas no changes were observed in the E2 fragments without putative binding sites (Fig. 5B). In line with this data, inhibition of HMGA1 could reduce the activity of each single mutant construct, but failed to decrease the luciferase activity of double mutant construct (Fig. 5C). Taken together, these data suggest that HMGA1 is bound to the RAD51 promoter and positively regulate transcription via two HMGA1 response elements.

### HMGA1 promotes DNA repair through the regulation of RAD51expression in CCA cells

**Fig. 6:**
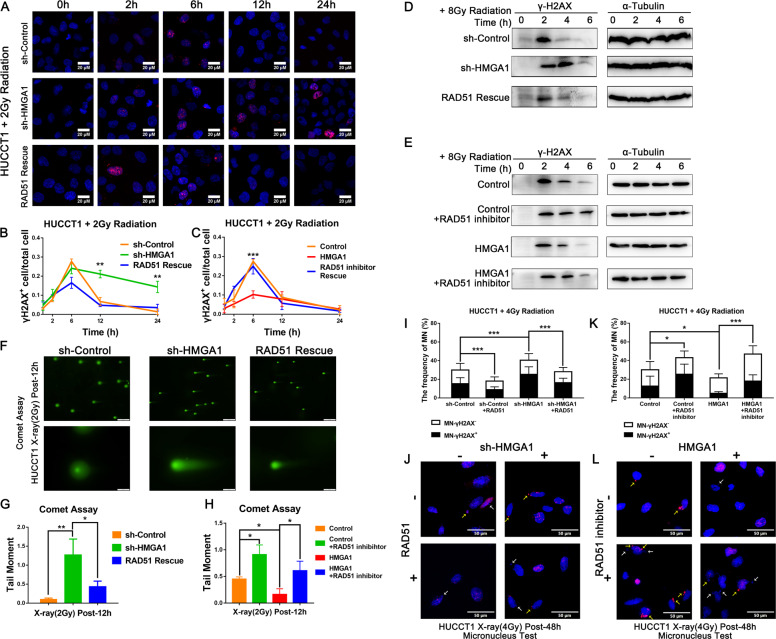
HMGA1 promotes DNA repair through the regulation of RAD51expression in CCA cells. **A**, **B** Immunofluorescence staining of γ-H2AX was measured in HUCTT1 cells transfected with sh-HMGA1, RAD51 and control vectors at different times (0 h, 2 h, 6 h, 12 h, and 24 h) after 2 Gy X-ray radiation. **C** Immunofluorescence staining of γ-H2AX was measured in HUCTT1 cells transfected with HMGA1 overexpression or control plasmid with or without RAD51 inhibitor (B02 9 μM) at different times (0 h, 2 h, 6 h, 12 h, and 24 h) after 2 Gy X-ray radiation. **D** Western blot analysis of γ-H2AX protein levels in HUCCT1 cells transfected with sh-HMGA1, RAD51 and control vectors at different times (0 h, 2 h, 4 h, and 6 h) after 8 Gy X-ray radiation. **E** Western blot analysis of γ-H2AX protein levels in HUCCT1 cells transfected with HMGA1 overexpression plasmid or control plasmid with or without RAD51 inhibitor (B02 9 μM) at different times (0 h, 2 h, 4 h, and 6 h) after 8 Gy X-ray radiation. **F**, **G** A comet assay was performed in HUCCT1 cells transfected with sh-HMGA1, RAD51 and control vectors at 12 h after 2 Gy X-ray radiation. **H** A comet assay was performed in HUCCT1 cells transfected with HMGA1 overexpression plasmid or control plasmid with or without RAD51 inhibitor (B02 9 μM) at 12 h after 2 Gy X-ray radiation. **I**, **J** The frequencies of MN in HUCCT1 cells transfected with sh-HMGA1, RAD51 and control vectors at 24 h after 4 Gy X-ray. **K**, **L** The frequencies of MN in HUCCT1 cells transfected with HMGA1 overexpression plasmid or control plasmid with or without RAD51 inhibitor (B02 9 μM) at 24 h after 4 Gy X-ray. Yellow arrows indicate MN-γH2AX^+^; white arrows indicate MN-γH2AX^−^. The significant differences between the two groups were analyzed by Student’s *t* test. *P < 0.05, **P < 0.01.

Since HMGA1 regulates RAD51 expression in CCA, we next wanted to determine whether HMGA1 is involved in DSB DNA repair in CCA cells. We first analyzed radiation-induced DNA damage repair by immunofluorescence quantification of γH2AX foci at different times (0-24 h) following irradiation with 2 Gy. Importantly, HMGA1 knockdown led to the decreased repair of radiation-induced DNA damage foci. In contrast, overexpression of RAD51 rescued the enhanced radiosensitivity induced by HMGA1 suppression in HUCCT1 cells (Fig. 6A, B and Fig. S3A). On the other hand, forced HMGA1 expression increased the repair of radiation-induced DNA damage foci and the effect could be reversed by RAD51 inhibitor treatment (B02 9 μM) (Fig. 6C, Figs. S3B, S4A). Subsequently, the expression of γH2AX was also measured by western blot. The results showed that the high level of γH2AX caused by HMGA1 knockdown was reduced by RAD51 overexpression (Fig. 6D). The overexpression of HMGA1 reduced the expression of γH2AX and RAD51 inhibitor (B02 9 μM) increase the γH2AX reduced by HMGA1 (Fig. 6E). Additionally, the comet assay showed that HMGA1 knockdown promoted comet tail formation and that the effect could be rescued by RAD51 overexpression (Fig. 6F, G). Forced HMGA1expression inhibit the comet tail formation and the effect could be reversed by RAD51 inhibitor treatment (B02 9 μM) (Fig. 6H, and Fig. S4B). Miconuleus (MN)-γH2AX^+^ are usually associated with DNA damage. To further strengthen the notion that HMGA1 promotes DNA repair through the regulation of RAD51 expression. We analyzed micronucleus frequency in the rescue experiments. As expected, HMGA1 knockdown led to increased frequencies of MN-γH2AX^+^ and overexpression of RAD51 significantly attenuated the effect. Fig. 6I, J and Fig. S4C). In contrast, overexpression of HMGA1 decreased the frequencies of MN-γH2AX^+^ which can be rescued by RAD51 inhibitor (B02 9 μM) (Fig. 6K, L and Fig. S4D). These results suggest that HMGA1 induces homologous recombination DNA repair by regulating RAD51 in CCA cells.

### HMGA1 promotes proliferation and invasion by increasing RAD51 level in CCA cells

**Fig. 7:**
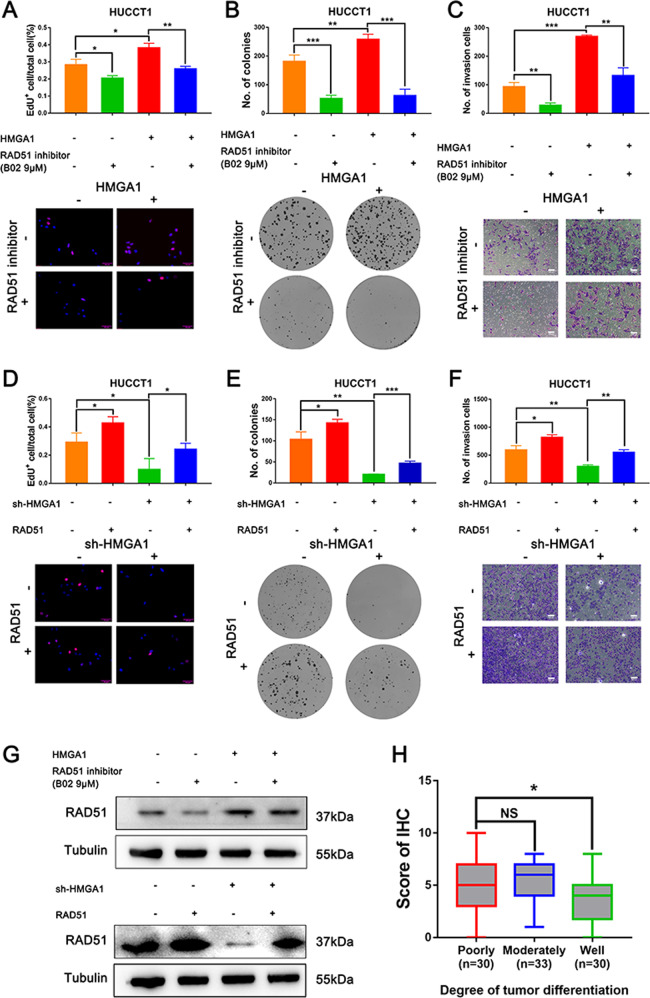
HMGA1 promotes proliferation and invasion by increasing RAD51 level in CCA cells. **A**, **B** The effect of RAD51 on the proliferation of CCA cells was examined by EdU and clonogenic assays of HUCCT1 cells. HUCCT1 cells were transfected with HMGA1 overexpression plasmid or control plasmid with or without RAD51 inhibitor (B02 9 μM) (*n* = 3 biologically independent samples). **C** Matrigel invasion data showing that inhibition of RAD51 decreased the invasion potential of CCA cells (*n* = 3 biologically independent samples). **D**, **E** EdU and clonogenic assays of HUCCT1 cells transfected with sh-HMGA1 or sh-Control with or without RAD51 overexpression (*n* = 3 biologically independent samples) showed RAD51 affect the proliferation of CCA cells. **F** Matrigel invasion data showing the potential of RAD51 to rescue the loss of HMGA1 in CCA cells as indicated (*n* = 3 biologically independent samples). **G** The protein levels of RAD51 were measured by western blot in HUCCT1 cells. **H** Statistical analysis of HMGA1 expression from IHC staining of our tissue microarray. High and low expression indicate the final score of each sample determined by two pathologists based on the intensity and extent of staining across the tissue microarray section. CCA patients were dichotomized into low (Score ≦7) and high (Score >7) RAD51 protein expression groups based on the IHC staining score. The high and low expression groups were separated based on the best cutoff. The *P*-value was obtained by Student’s *t* test. The results represent the mean ± S.D. of three independent experiments. *P < 0.05, **P < 0.01, ***P < 0.001.

To further investigate the functional significance of the HMGA1-RAD51 axis. We first performed rescue experiments by treating HMGA1-overexpressing and control cells with the RAD51 inhibitor B02. RAD51 expression was reduced both in control and HMGA1 overexpressed HUCCT1 cells upon B02 treatment. Functionally, the result of the EdU assay showed that the RAD51 inhibitor B02 significantly decreased the proliferation of CCA cells induced by HMGA1 (Fig. 7A and Fig. S5A). In accordance with the EdU data, the clonogenic assay showed that HMGA1-enhanced clonogenic capacity was attenuated in response to B02 treatment (Fig. 7B). In addition, a matrigel invasion assay was carried out, and the results demonstrated that the RAD51 inhibitor B02 decreased the invasive ability of HUCCT1 cells caused by HMGA1 overexpression (Fig. 7C). We next investigated whether enforced expression of RAD51 will rescue the effects induced by HMGA1 knockdown. To this end, RAD51 was overexpressed in HMGA1 knockdown HUCCT1 cells followed by functional assays. Intriguingly, RAD51 overexpression in HMGA1 knocked down cells could rescue the inhibitor effects of proliferation, clonogenic ability and invasiveness as measured by EdU, clonogenic and matrigel invasion assays (Fig. 7D–F and Fig. S5B). The rescue efficiency was detected by Western blot (Fig. 7G). To evaluate the clinical significance of RAD51 in CCAs, we performed immunohistochemistry in CCAs (*n* = 93) and analyzed the clinicopathologic parameters. As a result, high level of RAD51 was correlated with poorly-differentiated CCAs (Fig. 7H, and Fig. S5C). We further assessed the prognostic value, and the results of Kaplan–Meier survival analysis indicated that RAD51 expression had no significant influence on prognosis in CCA patients (Fig. S5D). We further analyzed the prognostic value of RAD51in CCAs in the TCGA-CCA cohort and found high level of RAD51 still showed no significant influence on overall survival (p = 0.16) disease-free survival (0.085) (Fig. S5E-F). These observations support the hypothesis that HMGA1 induces CCA cell proliferation and invasion through activation of RAD51.

## Discussion

Homologous recombination (HR) plays an essential role in maintaining genome integrity and stability [18]. Defects in HR repair contribute to genomic instability and lead to tumor development. On the other hand, overexpression of HR-related genes is associated with resistance to chemo- and radiotherapy [20]. RAD51 is the major strand transferase that plays a key role in HR repair [21]. RAD51 is elevated in many types of cancers, and overexpression of RAD51 has been linked to therapy resistance [22, 23]. In our study, we reported that the HMGA1 proto-oncogene was frequently upregulated in CCAs and was associated with a poor prognosis. Next, we provided evidence that HMGA1 transcriptionally activated RAD51 expression by directly binding to its promoter in CCA. Importantly, HMGA1-mediated RAD51 upregulation contributes to progression and radioresistance in CCA.

Previous studies showed that RAD51 expression was regulated by several transcription factors. HGF enhanced the transcription of RAD51 through IKZF1 and RUNX1 in human bone marrow mesenchymal stem cells [20]. Wild-type p63 and p73 induce RAD51 expression by binding to their promoter regions in MEFs [21]. CtBP1 (C-terminal binding protein 1) transactivates RAD51 and confers cisplatin resistance to breast cancer cells [22]. FOXM1 induces transcription of RAD51 in glioblastoma cells [23]. RAD51 expression is positively regulated by the EGR1 transcription factor [24]. Stat5a/b is a critical inducer of RAD51 and HR DNA repair in prostate cancer [25]. Conversely, wild-type p53 negatively regulates RAD51 by transcriptional repression [26]. The RAD51 promoter is highly active in cancer cells that lack functional p53 but less active in normal cells and in cancer cell lines with intact p53 function [27]. Our study identified HMGA1 as a novel transcription factor that induces RAD51 expression in cholangiocarcinoma.

RAD51 overexpression has been associated with a poor prognosis in several malignancies. Overexpression of RAD51 is a predictor of a poor outcome in colorectal adenocarcinoma [28], breast cancer [29], pancreatic cancer [30] and non-small cell lung cancer [31]. Overexpression of RAD51 stimulates homologous recombination and increases resistance to ionizing radiation [32]. RAD51 overexpression contributes to chemoresistance in human soft tissue sarcoma neuroblastoma [33].

In addition to conferring chemoresistance and radioresistance, high expression of RAD51 has been shown to exhibit oncogenic properties. RAD51 induces tumor growth and metastasis in esophageal squamous cell carcinoma [34]. RAD51 is overexpressed in high-grade serous ovarian cancer, and depletion of RAD51 results in G2/M arrest in ovarian cancer cells [35]. Our findings in this study demonstrated that HMGA1 knockdown impairs DNA repair by regulating RAD51 in CCA cells. Additionally, inhibition of RAD51 reverses HMGA1-driven CCA cell proliferation and invasion.

HMGA1 has been shown to be involved in multiple DNA repair pathways. HMGA1 promotes ATM expression and enhances cancer cell resistance to genotoxic agents [36]. Breast cancer cells overexpressing HMGA1 show a faster recovery upon induction of DNA DSBs via nonhomologous end-joining DNA repair [18]. On the other hand, HMGA1 downregulates the expression of BRCA1 and sensitizes MCF-7 cells to cisplatin-induced cell death [37]. HMGA1 inhibits the expression of nucleotide excision repair factor XPA [38]. In addition, HMGA1 is found in mitochondria, and HMGA1 overexpression inhibits mitochondrial base-excision repair upon oxidative damage. In this study, we found that HMGA1 transcriptionally activates RAD51 by binding to its promoter. HMGA1 knockdown impairs DNA repair in radiation-induced DNA damage. In this study, we provide the first evidence that HMGA1 is involved in homologous recombination repair through regulating RAD51 in cholangiocarcinoma.

In summary, we demonstrated that HMGA1 is frequently elevated in CCAs and that its high level is associated with a poor prognosis. Furthermore, HMGA1 transcriptionally activates RAD51 by binding to its promoter and it is involved in homologous recombination repair. HMGA1 knockdown sensitizes CCA cells to X-ray irradiation, and overexpression of RAD51 attenuates radiosensitivity. HMGA1 could represent a novel prognostic biomarker and a potential therapeutic target in CCA.

## Materials and methods

### Patients and tissue samples

This retrospective study included 93 cases of CCA and 3 cases of bile duct tissues that were collected in Qilu Hospital from 2010 to 2020. CCA specimens were obtained from primary CCA angiocarcinoma patients, and bile duct tissues were obtained from patients suffering from other benign pathologic changes. All participants in this study gave written informed consent as delineated by the protocol, which was approved by the Ethics Committee of Shandong University. Information about the clinical samples used in this study is supplied in Supplementary Table 1. A second cohort consisting of 67 CCA samples and 67 normal bile duct samples was obtained to verify the expression level of HMGA1 from GSE76297 [39]. The third cohort consisting of 36 CCA samples and 9 normal bile duct samples was obtained from The Cancer Genome Atlas (TCGA) dataset.

### Immunohistochemistry

Immunohistochemistry (IHC) staining was performed on formalin-fixed paraffin-embedded CCA samples and xenograft tumors. Briefly, tissue slides were deparaffinized and rehydrated in a graded series of ethanol. After that, antigenic retrieval proceeded by microwave heating. Nonspecific antigens were blocked with 1.5% normal goat serum. Then, they were incubated at 4 °C in a moist chamber overnight with antibodies against human HMGA1, RAD51 and Ki67. The next day, the slides were incubated with the corresponding secondary antibody. The staining was detected with a DAB detection system based on the Biotin-Streptavidin HRP Detection Systems (Zhongshan Biotechnology Company, China). The final score of each sample was assessed by two independent pathologists based on the intensity and extent of staining across the section.

### Cell lines and cell culture

HUCCT1 and QBC939 cells were obtained from Professor Han Bo’s laboratory in the Department of Pathology, Shandong University of Basic Medical Sciences. HCCC-9810 cells were purchased from the Cell Bank of the Chinese Academy of Sciences (Shanghai, China). HEK293T cells were purchased from the American Type Culture Collection (ATCC, VA, USA). HUCCT1, QBC939 and HCCC9810 cells were routinely cultured in RPMI 1640 medium (Gibco, NY, USA). The HEK293T cells were cultured in DMEM (Gibco, NY, USA). All media were supplemented with 10% FBS (Gibco, NY, USA), 100 U/ml penicillin and 100 μg/ml streptomycin. All of the cells were cultured at 37 °C and 5% CO_2_ in a humidified incubator (Thermo Fisher Scientific, IL, USA).

### Constructs and lentivirus infection

The HMGA1 overexpression plasmid was purchased from Gikai Gene Company (GV492, Shanghai, China). The Tet-on-sh-HMGA1 shRNA vector (pZIP-TRE3G-ZsGreen-Puro) was constructed by Vigene Biosciences (Jinan, Shandong, China) based on the si-HMGA1 sequence. The PCMV-RAD51 plasmid was obtained from Changshun Shao’s laboratory in the Ministry of Education and Department of Molecular Medicine and Genetics, Shandong University School of Basic Medical Sciences. Lentivirus was produced in HEK293T cells. For stable infection, 1 × 10^5^ cells were plated in 6-well plates with 2 ml medium without antibiotics. After overnight incubation, the medium was replaced by 1 ml Opti-MEM Reduced-Serum Medium (Gibco, NY, USA) containing 50 μl of concentrated lentiviral particles and 8 μg/ml of polybrene per well. Fresh medium containing 2 μg/ml puromycin (Invitrogen, USA) was added to each well 24 h later and then continued for two weeks. The sequence of shRNA is shown in Supplementary Table 3.

### RNA-seq

Three samples of HUCCT1 cells transfected with shHMGA1 or control vector were subjected to RNA-seq. Total RNA was extracted using TRIzol reagent (Invitrogen, CA, USA) following the manufacturer’s procedure. The total RNA quantity and purity were analyzed with a Bioanalyzer 2100 and RNA 1000 Nano LabChip Kit (Agilent, CA, USA) with RIN number >7.0. Poly(A) RNA was purified from total RNA (5 µg) using poly-T oligo-attached magnetic beads using two rounds of purification. Following purification, the mRNA was fragmented into small pieces using divalent cations at an elevated temperature. Then, the cleaved RNA fragments were reverse transcribed to create the final cDNA library in accordance with the protocol for the mRNA Seq sample preparation kit (Illumina, San Diego, USA), and the average insert size for the paired-end libraries was 300 bp (±50 bp). Then, we performed paired-end sequencing on an Illumina X10 at LC Sciences (USA) following the vendor’s recommended protocol. The RNA-seq data generated in this study have been deposited in the NCBI GEO database under the accession number GSE163759.

### Bioinformatic analysis

The differentially expressed mRNAs in the TCGA data were selected with log2 (fold change) >2 or log2 (fold change) <−2 and with statistical significance (*p* < 0.05) by the limma package of R. The differentially expressed mRNAs and genes of RNA-seq data were selected with log2 (fold change) >0.3 or log2 (fold change) <−0.3 and with statistical significance (*p* < 0.05) by the DEseq package of R. The GESA analysis was performed by GSEA software v. 3.0, using the local human-KEGG database and log2 ratio of classes method. KEGG pathways in CCA and normal tissue with significant enrichment results were demonstrated based on the NES (net enrichment score), gene ratio and *P*-value. The heatmap was generated by R package pheatmap. The R package statistics were used for the cluster computation and Z-score normalization of the data. We applied Gene Ontology (GO) classification to uncover the functions of intersecting genes and further tested the biological links to coregulated genes. The R package GOplot was used to integrate the quantitative information by implementing novel and high-quality plotting. The K–M curves of the CCA patients and the pancancer analysis of HMGA1 were conducted with GEPIA [40].

### Western blot analysis

Cells were lysed on ice with lysis buffer (Sangon Biotech). The protein concentration was determined with the BCA Protein Assay Kit (Beyotime Institute of Biotechnology). Protein samples were separated by SDS-PAGE and electrotransferred to PVDF membranes. The membranes were blocked by incubating in 5% skimmed milk for 1 h and the incubated overnight with primary antibodies at 4 °C. Proteins of interest were detected with HRP-labeled secondary antibodies and the ECL system (GE Healthcare). α-Tubulin was used as an endogenous control. All antibody information is listed in Supplementary Table 4.

### RNA isolation and real-time PCR

Total RNA from the cells was extracted with TRIzol reagent (Invitrogen, USA) according to the manufacturer’s protocol. RNA was reverse transcribed to cDNA using the PrimeScript RT reagent Kit (Takara, JAPAN). Real-time PCR was performed with SYBR Green mix and detected by the Bio-Rad CFX96. GAPDH served as an endogenous control. The primer sequences are listed in Supplementary Table 3.

### EdU assay

Cells were seeded on glass coverslips in 24-well plates at densities of 4–10 × 10^4^ cells per well. The EdU assay was performed using an EdU Kit (RiboBio, Guangzhou, China) following the manufacturer’s instructions. Briefly, the cells were incubated in cell culture medium containing EdU for 0.5–1 h. Then, the cells were fixed and stained with Apollo fluorescent dye and Hoechst 33342. The glass coverslips were fixed on glass slides and captured under a fluorescence microscope.

### Clonogenic assay

The cells were seeded in a six-well plate (500–2000 cells per well) and cultured for 1–2 weeks. The colonies were fixed with methanol and stained with 0.1% crystal violet, and the number of colonies containing more than 50 cells was counted. The data presented are the mean ± S.E. and represent three independent experiments.

### Matrigel invasion assay

Invasion and migration assays were performed in a 24-well Transwell chamber (BD Biosciences, USA) system with 8 μm pores coated with diluted Matrigel (BD Biosciences, USA). Briefly, 1.5–2 × 10^5^ cells were seeded into the upper chambers in media containing no FBS, and the lower chambers were filled with culture media containing 20% FBS as a chemoattractant. After incubating at 37 °C for 12 to 48 h depending on cell lines, cells that penetrated through the membrane were fixed with methanol and stained with crystal violet.

### X-ray radiation

X-rays were generated with an X-RAD 225 OptiMAX X-ray biological irradiator (PXi, USA) operated at 225 kV and 17.8 mA. The dose rates were 1.1 Gy/min. Cells in exponential growth phases were irradiated at room temperature, and nonirradiated culture cells, which were handled in parallel with the irradiated samples, were used as controls.

### Comet assay

The comet assay was performed according to the manufacturer’s protocol (Trevigen #4250-050-K). The comet assay 2-well ES unit with a starter kit was used to perform the assay. The TriTek CometScore (Version 1.5.2.6) was used to analyze the images. At least 50 cells were analyzed and plotted using prism (GraphPad Software). Student’s *t* test was used to test the statistical significance.

### Immunofluorescence

Cells were seeded on sterile glass coverslips and cultured overnight at 37 °C. The cells were washed with PBS and fixed for 20 min with 4% paraformaldehyde. Following blocking with 10% goat serum for 2 h at room temperature, the cells were incubated with primary antibodies (anti-γH2AX) overnight at 4 °C. The cells were then incubated with rhodamine/FITC-labeled secondary antibody (Beyotime Biotechnology, China) for 1 h at room temperature in the dark. The cells were washed with PBS three times before counterstaining with 1 µg/ml DAPI for 5 min in the dark. Finally, the slides were mounted with antifading reagent and examined with a fluorescence microscope (Olympus, Japan).

### Chromatin immunoprecipitation (ChIP)

ChIP assays were conducted using an EZ-Magna ChIP A/G Chromatin Immunoprecipitation Kit (Millipore) following the manufacturer’s instructions. Briefly, the cells were cross-linked using formaldehyde. Then, the cells were lysed, and the DNA was sheared to 200–500 bp fragments by sonication. The cell lysate was incubated with the HMGA1 antibody and magnetic beads overnight at 4 °C with rotation. The DNA was purified and analyzed by qRT-PCR.

### Luciferase assay

RAD51 promoters of different lengths were cloned into the pGL4 plasmid (Promega). Then, the RAD51 promoter reporter vector was cotransfected with pRL-TK plasmids into HUCCT1 cells with stable expression of tet-on shRNA or control vector. Luciferase activity was measured 24 h after transfection using the Dual-Luciferase Reporter Assay System (Promega) according to the manufacturer’s protocol.

### Xenograft tumor model

Male BALB/c nude mice (4–6-week-old, 5 or 6 mice for each group) were randomly divided into two groups and were injected subcutaneously into the left underarm with cell suspensions (5 ×10^6^ cells in 100 µl PBS). The tumor size was measured every three days after 1 week, and the mice were killed with anesthesia after two weeks. The tumor volume was calculated according to the formula TV (cm^3^) =*a* × *b*^2^ × π/6, where ‘*a*’ is the longest diameter and ‘*b*’ is the shortest diameter. Hematoxylin and eosin (H/E) staining and immunohistochemistry were performed on sections from embedded samples. All animal experiments were performed with the approval of the Shandong University Animal Care and Use Committee.

### Statistics analysis

Student’s *t* test and one-way ANOVA were used to determine significance. The results represent the mean ± S.D. of three independent experiments. *P*-values <0.05 were significant.

## Supplementary information

Supplementary Figure 1

Supplementary Figure 2

Supplementary Figure 3

Supplementary Figure 4

Supplementary Figure 5

Supplementary Figure Legends

Supplementary Tables

## Data Availability

The data used to support the findings of this study are available from the corresponding author upon request.
