# Supplementary figures and images for: Overexpression of HMGA1 confers radioresistance by transactivating RAD51 in cholangiocarcinoma
Source: Cell Death Discov. 2021 Oct 29;7:322. doi: 10.1038/s41420-021-00721-8 (PMC8556338; doi:10.1038/s41420-021-00721-8)

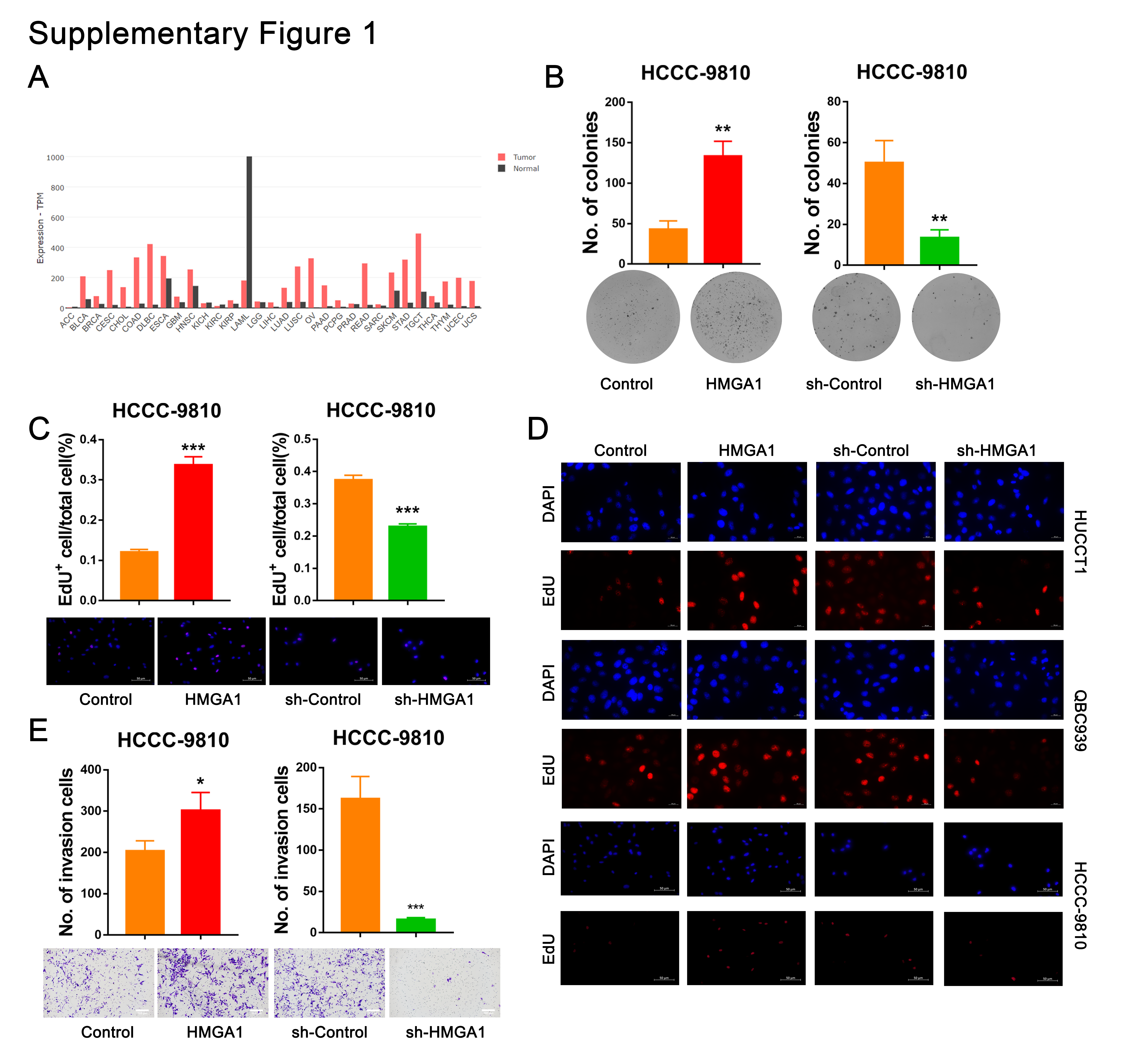

Supplement: Supplementary file 1 — Supplementary Figure 1 [file 41420_2021_721_MOESM1_ESM.tif]

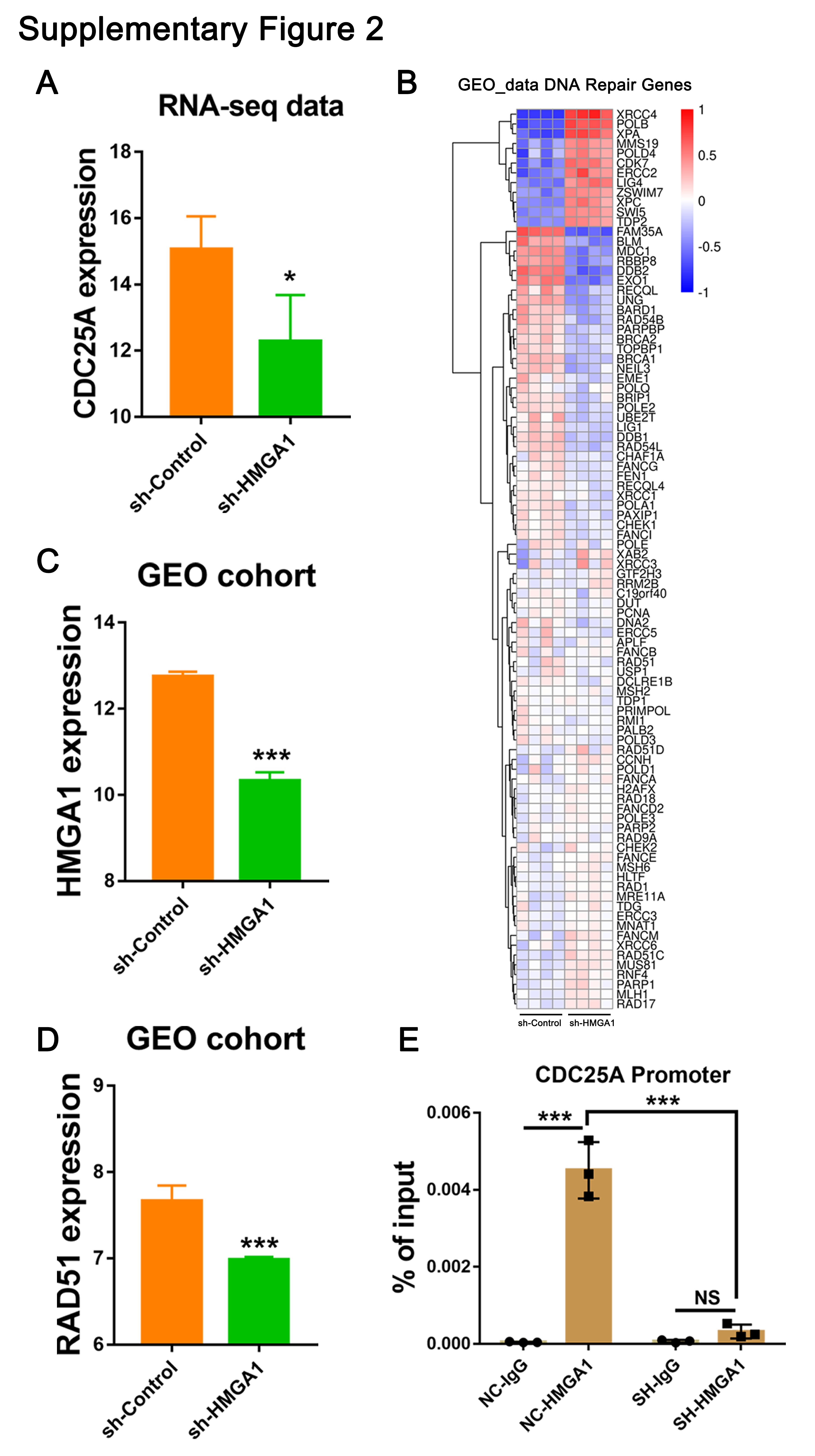

Supplement: Supplementary file 2 — Supplementary Figure 2 [file 41420_2021_721_MOESM2_ESM.tif]

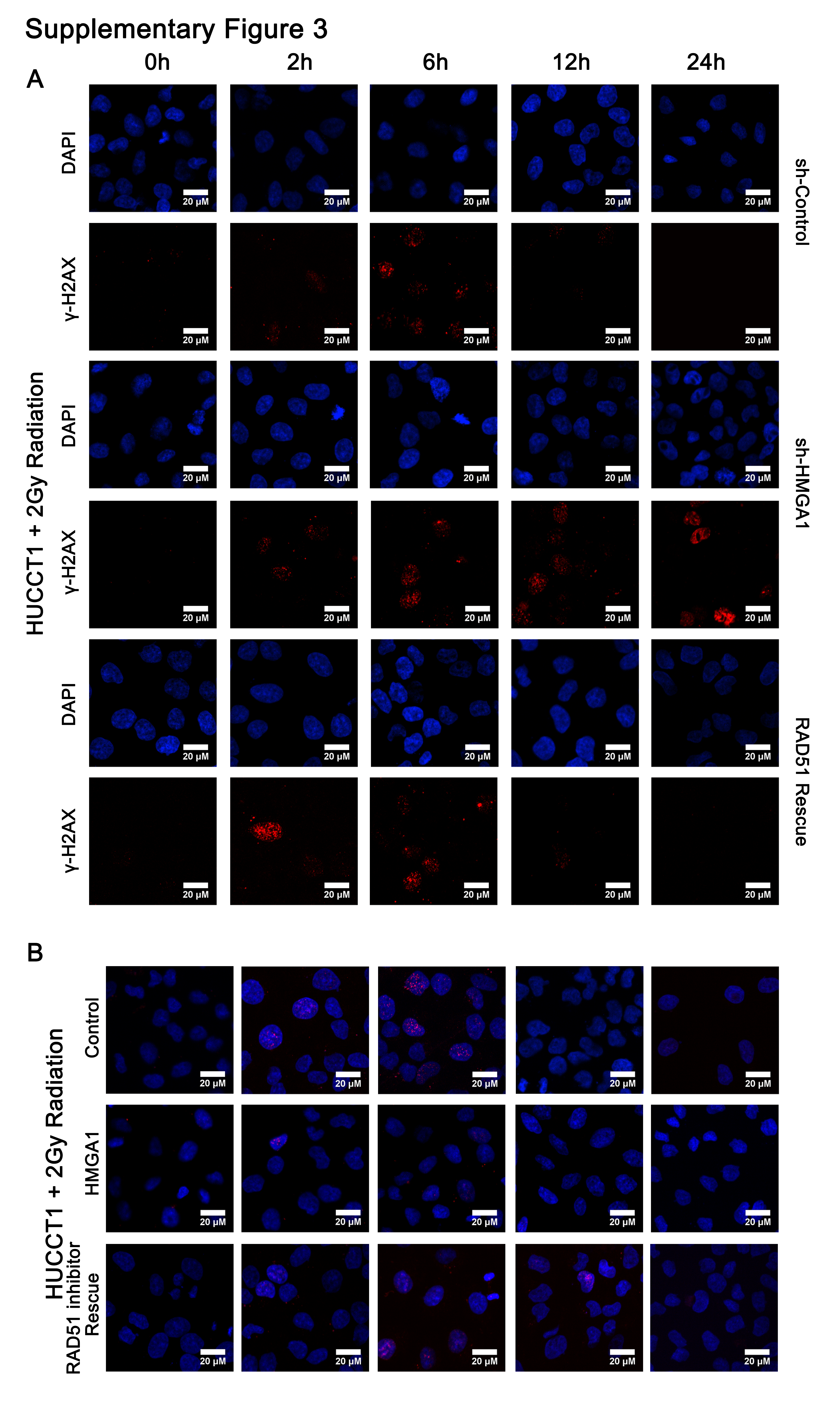

Supplement: Supplementary file 3 — Supplementary Figure 3 [file 41420_2021_721_MOESM3_ESM.tif]

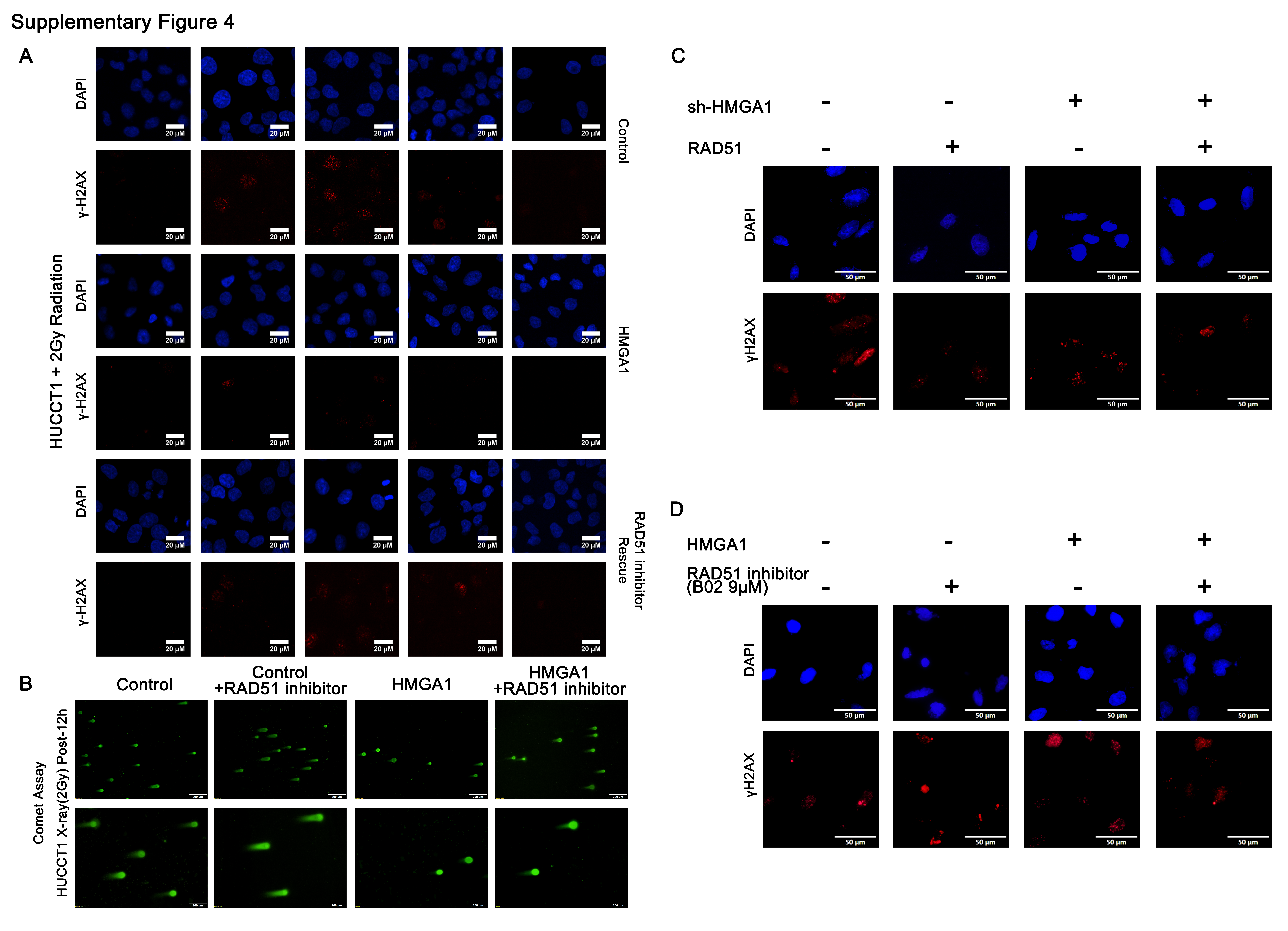

Supplement: Supplementary file 4 — Supplementary Figure 4 [file 41420_2021_721_MOESM4_ESM.tif]

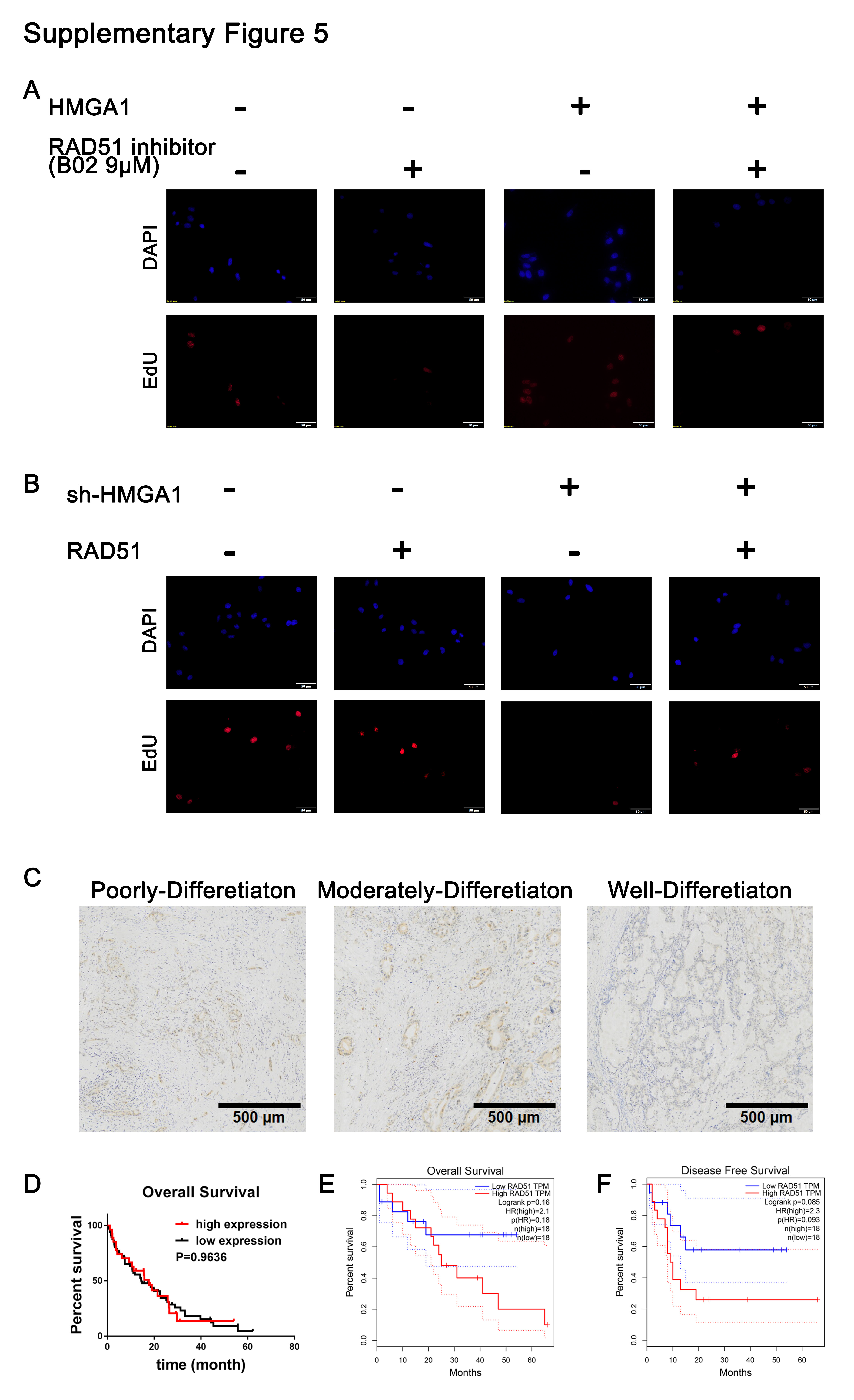

Supplement: Supplementary file 5 — Supplementary Figure 5 [file 41420_2021_721_MOESM5_ESM.tif]
