# Supplementary material for: Overexpression of HMGA1 confers radioresistance by transactivating RAD51 in cholangiocarcinoma
Source: Cell Death Discov. 2021 Oct 29;7:322. doi: 10.1038/s41420-021-00721-8 (PMC8556338; doi:10.1038/s41420-021-00721-8)
Supplement: Supplementary file 6 — Supplementary Figure Legends [file 41420_2021_721_MOESM6_ESM.doc]

**Supplementary Figure Legends**

**Supplementary Figure 1**

**A,** The pan-cancer analysis revealed the expression of HMGA1 in different cancers from <http://gepia.cancer-pku.cn/index.html.> **B-C,** clonogenic and EdU assays were performed to measure the effect of HMGA1 on cell proliferation of HCCC-9810 cells (n = 3 biologically independent samples). **D,** Related to Fig.2-B EdU assays of CCA cells with HMGA1 overexpression (HMGA1) or knock down (sh-HMGA1) compared to corresponding control (n = 3 biologically independent samples). **E,** Matrigel invasion assay were performed to measure the effect of HMGA1 on invasion capacity of HCCC-9810 cells (n = 3 biologically independent samples). P value was obtained by Student’s t-test. Results represent the mean ± S.D. of three independent experiments. *P < 0.05, **P < 0.01, ***P < 0.001.

**Supplementary Figure 2**

**A,** CDC25A was one of the target gene of HMGA1. The expression of CDC25A was decreased in HUCCT1 cells with knockdown of HMGA1. **B,** 92 differently expressed DNA repair genes (originate from https://www.mdanderson.org/documents/Labs/Wood-Laboratory/human-dna-repair-genes.html) were filtered from GSE35525 dataset (MDA-MB-231 cells with or without knockdown of HMGA1, log2 (fold change) >0.3 or log2 (fold change) <-0.3 and with statistical significance (p value < 0.05)). **C-D,** The expression of HMGA1 and RAD51 was decreased in MDA-MB-231 cells (GSE35525) with knockdown of HMGA1. **E,** qPCR analysis of ChIP samples showed the enrichment of CDC25A promoter region which was as a positive control. P value was obtained by Student’s t-test. Results represent the mean ± S.D. of three independent experiments. *P < 0.05, **P < 0.01, ***P < 0.001.

**Supplementary Figure 3**

**A,** Related to Fig.6A. Immunofluorescence staining of γ-H2AX was measured in HUCTT1 cells transfected sh-HMGA1 and RAD51 overexpression plasmids as indicated radiated with Gy X-ray at different times (0 h, 2 h, 6 h, 12 h, and 24 h). **B**, Immunofluorescence staining of γ-H2AX was measured in HUCTT1 cells transfected with HMGA1 overexpression plasmid or control plasmid with or without RAD51 inhibitor (B02 9 μM) at different times (0 h, 2 h, 6 h, 12 h, and 24 h) after 2 Gy X-ray radiation.

**Supplementary Figure 4**

**A**, Immunofluorescence staining of γ-H2AX was measured in HUCTT1 cells transfected with HMGA1 overexpression plasmid or control plasmid with or without RAD51 inhibitor (B02 9 μM) at 12 h after 2 Gy X-ray radiation. **B**, Comet assay was performed in HUCCT1 cells transfected with HMGA1 overexpression plasmid or control plasmid with or without RAD51 inhibitor (B02 9 μM) at 12 h after 2 Gy X-ray radiation. **C,** Related to f Fig.6J. The frequencies of MN in HUCCT1 cells transfected with sh-HMGA1, RAD51 and control vectors at 24 h after 4 Gy X-ray. **D,** Related to Fig.6L. The frequencies of MN in HUCCT1 cells transfected with HMGA1 overexpression plasmid or control plasmid with or without RAD51 inhibitor (B02 9 μM) at 24 h after 4 Gy X-ray.

**Supplementary Figure 5**

**A-B,** Related to Fig.7-A and Fig.7-D. EdU assays of HUCCT1 cells. HUCCT1 cells were transfected with HMGA1 overexpression plasmid or control plasmid with or without RAD51 inhibitor (BO2 9μM) (n = 3 biologically independent samples). The other group of HUCCT1 cells were transfected with sh-HMGA1 or sh-Control with or without RAD51 overexpression. **C**, Representative images of immunohistochemical (IHC) staining of RAD51 in our tissue microarray (containing 93 samples of CCA tissues collected from Qilu Hospital, Shandong University). **D,** Kaplan–Meier analysis of the correlation between RADR51 expression and clinical prognosis based on data from our tissue microarray. **E-F**, Kaplan–Meier analysis of the correlation between RAD51 expression and the clinical prognosis (http://gepia.cancer-pku.cn/index.html).
