# Supplementary material for: Overexpression of HMGA1 confers radioresistance by transactivating RAD51 in cholangiocarcinoma
Source: Cell Death Discov. 2021 Oct 29;7:322. doi: 10.1038/s41420-021-00721-8 (PMC8556338; doi:10.1038/s41420-021-00721-8)
Supplement: Supplementary file 7 — Supplementary Tables [file 41420_2021_721_MOESM7_ESM.docx]

**Supplementary Table 1.** Clinicopathological characteristics of patients from Qilu Hospital enrolled in this study

| **Title** | **Parameters** | **Scale and range** |
| --- | --- | --- |
| Age | Range | 41-83 |
|  | Mean±SD | 61.2±8.4 |
|  | Median | 61 |
| Gender | Male | 60 |
|  | Female | 33 |
| Lymph node metastases | Absent | 73 |
|  | Present | 20 |
| Follow-up | Alive with disease | 21 |
|  | Death | 72 |
|  | Loss of follow-up | 0 |
| CA125 | ＜500 | 64 |
|  | ≥500 | 20 |
|  | Unknown | 9 |
| Degree of tumor differentiation | Poorly | 30 |
|  | Moderately | 33 |
|  | Well | 30 |

**Supplementary Table 2.** Correlation between HMGA1 expression and clinicopathological characteristics

| **Clinicopathological features** | | **HMGA1 expression** | | **P-value** |
| --- | --- | --- | --- | --- |
|  |  | **Low expression** | **High expression** |  |
| Age | ＜60 | 22 | 15 | 0.9593 |
|  | ≥60 | 33 | 23 |  |
| Gender | Male | 35 | 25 | 0.8311 |
|  | Female | 20 | 13 |  |
| CA199 | ＜500 | 41 | 23 | 0.0219 |
|  | ≥500 | 7 | 13 |  |
| Lymph node metastasis | Absent | 47 | 26 | 0.0494 |
|  | Present | 8 | 12 |  |
| Degree of tumor differentiation | Poorly | 7 | 23 | <0.0001 |
|  | Moderately | 22 | 11 |  |
|  | Well | 26 | 4 |  |

**Supplementary Table 3.** Primer sequences

| method | Prime name | Primer sequence (5’-3’) |
| --- | --- | --- |
| qPCR | HMGA1-F | CAACTCCAGGAAGGAAACCA |
| qPCR | HMGA1-R | AGGACTCCTGCGAGATGC |
| qPCR | RAD51-F | CAACCCATTTCACGGTTAGAGC |
| qPCR | RAD51-R | TTCTTTGGCGCATAGGCAACA |
| qPCR | GAPDH-F | TGCACCACCAACTGCTTAGC |
| qPCR | GAPDH-R | GGCATGGACTGTGGTCATGAG |
| sh-RNA | sh-HMGA1 | ACAACTCCAGGAAGGAAACCAA |
| ChIP-PCR | Site1(F) | TGCCTCAGCCTCCCAAAG |
| ChIP-PCR | Site1(R) | GCTCAAGCCTGTAATCCC |
| ChIP-PCR | Site2(F) | CTCAGCCTTTCGAGTAGC |
| ChIP-PCR | Site2(R) | TCAGGGGTTGGAGATAAA |
| ChIP-PCR | CDC25A(F) | ACACTGGGAACAGTGTCAAGTGCA |
| ChIP-PCR | CDC25A(R) | ACCACTTCTTAGCCCCCTGGGA |

**Supplementary Table 4.** Primary and secondary antibodies

| Primary antibodies | Host | Dilution | Manufacture | Usage |
| --- | --- | --- | --- | --- |
| HMGA1 | Rabit | 1:400 | ab4078 - Abcam | Immunohistochemistry |
| Ki67 | Rabit | 1:400 | #9129 - Cell signaling | Immunohistochemistry |
| HMGA1 | Rabit | 1:1000 | ab4078 - Abcam | Western Blot |
| Rad51 | Rabit | 1:1000 | ab111534 - Abcam | Western Blot |
| γH2AX (Ser139) | Rabit | 1:1000 | ab11174 - Abcam | Western Blot |
| α-Tubulin | Mouse | 1:5000 | 66031-1-Ig - Proteintech | Western Blot |
| γH2AX (Ser139) | Mouse | 1:200 | 3135384 - Millipore | Immunofluorescence |
| HMGA1 | Rabit | 1:10 | ab252930 - Abcam | ChIP, Co-IP |
| Goat anti-rabbit IgG-HRP conjugated | | 1:10000 | 115-035-003 - Jackson |  |
| Goat anti-mouse IgG-HRP conjugated | | 1:10000 | 111-035-003 - Jackson |  |

**Inserted sequence of HMGA1 overexpression plasmid**

ATGAGTGAGTCGAGCTCGAAGTCCAGCCAGCCCTTGGCCTCCAAGCAGGAAAAGGACGGCACTGAGAAGCGGGGCCGGGGCAGGCCGCGCAAGCAGCCTCCGGTGAGTCCCGGGACAGCGCTGGTAGGGAGTCAGAAGGAGCCCAGCGAAGTGCCAACACCTAAGAGACCTCGGGGCCGACCAAAGGGAAGCAAAAACAAGGGTGCTGCCAAGACCCGGAAAACCACCACAACTCCAGGAAGGAAACCAAGGGGCAGACCCAAAAAACTGGAGAAGGAGGAAGAGGAGGGCATCTCGCAGGAGTCCTCGGAGGAGGAGCAG

**Inserted sequence of FL luciferase reporters**

GCGTGATCTCGCTCACTGCAACCTCCACCTCCCGGGTTCAAGCACTTCTCTGCCTCAGCCTCCCAAAGAGCTGGGATTACAGGCATGCACCACCACGCCCGGCTAATTTTTGTAGTTTTAGTAGAGATGGGGTTTTGCCATCTTGGCCAGGCTGGTCTTGAACTCCTGACCTCGTGATCCGCCCACCTTGGCCTCCCAAAGTTCTGGGATTACAGGCTTGAGCCACCGCGCCTGGCCTCTCCTTACATGTTTTGTTGTTGTCGCTTGTCTTTTGAGACAAGGTCTCACTCTGTAGCCCAGGCTGGAGTGCAGTGGCCCATCATAGCTCACTGCAGCCTTGACGTCCTGAGCTCAAGCAGTCCTCCCACCTCAGCCTCCGGGATAGCTGAGACCACAGGCACAAGCCACCACGCCCAGCTTATTTTTTTTTTTTTTTTTTTTGAGATATGGGGTCCCACTATGTTGCCCAGGCTGGTCTCCAACTCCTGAGCTCAAGCGATCCTCCCCCACCTTGGCTTCCCAAAGTGCTGGGATTACAGGTGTGAGCCACCGTGCAGGCCTTATATGATCTTCATATCCTGAACTAAATGTAACCTTCCAGTTTCGGCACTTGCTCTGGCACTTTTCCTCCCTCGCCAGATAATACTAATCTTTAATCATGTAGTTCGTTTCCATGCCCATACTACCCTATTTGCTTATAATGTCTTCCACTTCGCCCAAGAATCCCTACTCAGCTAGCTTGTGGTGTTGTTTTGACACAGTCTCGCTCTGTCGCCCAGGCTGGAGTACAGCGGCGAGATCTCGGTTGGCTGCAACCTCCTCCTGAGTTCAAGCGATTCTCATGCCTCAGCCTTTCGAGTAGCTAGGATTACAGGCATGTGCCACAAAACCTGGCTAATTTTTGTATTTTTTACTAAAGACGAGGTTTCACCACGTTGGCCAGGTTTATCTCCAACCCCTGACCTCAGGTGATCCGCCTGCCTTGGCCTCCTAAACTGCTGGGATTACTGGCGTGAACCACCGCGCCCGGCCCTACTCAGCCTTTAAAACCGGAATCACGGGTCAAAACTTTCTGGTAAACCACGATACGGTTTAGGTTATGAAATTCAATGCCCCCTTCCTCTGAACTCCTGCAAATCTCCAGTAAAGCACCACAGATTGACGAATATTCCAGCCATTTCCCTCTCCCGTACGCTAGCTCCATTTCCCACTTCTATCCATCTTCTCGAGCTTCCTCAGCTCCTCCACCTCCATGAGGCCTGGAAAGCACCTTGCTCCAGGAATGCGAGTAGGAGGCTCAGAGCGACCAGAAGTGCCAAAAGCTGACATTCAGATACTGCCGAAACAAACCACAAGAGCGCTAGGGCCCCCGCTAATAGTCCAGCTGCGATGGTGAGAACTCGCGGACCCGCCGGCGATGCATGCCGGGAGATGTAGTCCCGGGCCGACGCATTACCTCTTGGGAGTCGTGGTCTTCGATCTGGTAAACAGAAGACGGCAACTCGGTTAAGTCTTCCCCCACCGCCCCCTGAAATCCCTCGCCCCACCCGCGAGGGACTGGGGTAGGAGTAGGGGCGTTGCCGTGGTTAGCCTCGAACTCCTAGGCTCAGACGATACTCTCGCCTCGGCCTCCCGAGCAGCTGGGACTACACGCGTGAGCCACCGCCCCCGGCATAAAGTTTGAATTAGTCCTTACGCAAAAAGGGAAGAGGGCAGTCTGTAAACTCGCGCAGGATCAAGCTCTCGAGCTCCCGTCTTGGGTTAGCGCGCAGGGCGGAAGCGGGGAGAAGGCGGATCCGGGAGGCGGGGATACGTTACGTCGACGCGGGCGTGACCCTGGGCGAGAGGGTTTGGCGGGAATTCTGAAAGCCGCTGGCGGACCGCGCGCAGCGGCCAGAGACCGAGCCCTAAGGAGAGTGCGGCGCTTCCCGAGGCGTGCAGCTGGGAACTGCAACTCATCTGGGTTGTGCGCAGAAGGCTGGGGCAAGCGAGTAGAGAAGTGGAGCGTAAGCCAGGGGCGTTGGGGGCCGTGCGGGTCGGGCGCGTGCCACGCCCGCGGGGTGAAGTCGGAGCGCGGGGCCTGCTGGAGAGAGGAGCGCTGCGGACCGAGGTGAGTGTGTGAGGCGCAGGCTGGGCCCTCCAGAGCCGCGGCTCGTCCTCGCCCACCTGCGTCCTGGCCGGTCCAGTGCTCAGCGGCAGTTGGGGCCTCCGCGCGCAGTGTGAAACCCGGACGTGGCAGGGCGTGTCCGCGCCCGACCGACCCTCAGCTGCTGGGGCGAAAACACAAGTGGACCTCAGTC

**Inserted sequence of E1 luciferase reporters**

GTAGAGATGGGGTTTTGCCATCTTGGCCAGGCTGGTCTTGAACTCCTGACCTCGTGATCCGCCCACCTTGGCCTCCCAAAGTTCTGGGATTACAGGCTTGAGCCACCGCGCCTGGCCTCTCCTTACATGTTTTGTTGTTGTCGCTTGTCTTTTGAGACAAGGTCTCACTCTGTAGCCCAGGCTGGAGTGCAGTGGCCCATCATAGCTCACTGCAGCCTTGACGTCCTGAGCTCAAGCAGTCCTCCCACCTCAGCCTCCGGGATAGCTGAGACCACAGGCACAAGCCACCACGCCCAGCTTATTTTTTTTTTTTTTTTTTTTGAGATATGGGGTCCCACTATGTTGCCCAGGCTGGTCTCCAACTCCTGAGCTCAAGCGATCCTCCCCCACCTTGGCTTCCCAAAGTGCTGGGATTACAGGTGTGAGCCACCGTGCAGGCCTTATATGATCTTCATATCCTGAACTAAATGTAACCTTCCAGTTTCGGCACTTGCTCTGGCACTTTTCCTCCCTCGCCAGATAATACTAATCTTTAATCATGTAGTTCGTTTCCATGCCCATACTACCCTATTTGCTTATAATGTCTTCCACTTCGCCCAAGAATCCCTACTCAGCTAGCTTGTGGTGTTGTTTTGACACAGTCTCGCTCTGTCGCCCAGGCTGGAGTACAGCGGCGAGATCTCGGTTGGCTGCAACCTCCTCCTGAGTTCAAGCGATTCTCATGCCTCAGCCTTTCGAGTAGCTAGGATTACAGGCATGTGCCACAAAACCTGGCTAATTTTTGTATTTTTTACTAAAGACGAGGTTTCACCACGTTGGCCAGGTTTATCTCCAACCCCTGACCTCAGGTGATCCGCCTGCCTTGGCCTCCTAAACTGCTGGGATTACTGGCGTGAACCACCGCGCCCGGCCCTACTCAGCCTTTAAAACCGGAATCACGGGTCAAAACTTTCTGGTAAACCACGATACGGTTTAGGTTATGAAATTCAATGCCCCCTTCCTCTGAACTCCTGCAAATCTCCAGTAAAGCACCACAGATTGACGAATATTCCAGCCATTTCCCTCTCCCGTACGCTAGCTCCATTTCCCACTTCTATCCATCTTCTCGAGCTTCCTCAGCTCCTCCACCTCCATGAGGCCTGGAAAGCACCTTGCTCCAGGAATGCGAGTAGGAGGCTCAGAGCGACCAGAAGTGCCAAAAGCTGACATTCAGATACTGCCGAAACAAACCACAAGAGCGCTAGGGCCCCCGCTAATAGTCCAGCTGCGATGGTGAGAACTCGCGGACCCGCCGGCGATGCATGCCGGGAGATGTAGTCCCGGGCCGACGCATTACCTCTTGGGAGTCGTGGTCTTCGATCTGGTAAACAGAAGACGGCAACTCGGTTAAGTCTTCCCCCACCGCCCCCTGAAATCCCTCGCCCCACCCGCGAGGGACTGGGGTAGGAGTAGGGGCGTTGCCGTGGTTAGCCTCGAACTCCTAGGCTCAGACGATACTCTCGCCTCGGCCTCCCGAGCAGCTGGGACTACACGCGTGAGCCACCGCCCCCGGCATAAAGTTTGAATTAGTCCTTACGCAAAAAGGGAAGAGGGCAGTCTGTAAACTCGCGCAGGATCAAGCTCTCGAGCTCCCGTCTTGGGTTAGCGCGCAGGGCGGAAGCGGGGAGAAGGCGGATCCGGGAGGCGGGGATACGTTACGTCGACGCGGGCGTGACCCTGGGCGAGAGGGTTTGGCGGGAATTCTGAAAGCCGCTGGCGGACCGCGCGCAGCGGCCAGAGACCGAGCCCTAAGGAGAGTGCGGCGCTTCCCGAGGCGTGCAGCTGGGAACTGCAACTCATCTGGGTTGTGCGCAGAAGGCTGGGGCAAGCGAGTAGAGAAGTGGAGCGTAAGCCAGGGGCGTTGGGGGCCGTGCGGGTCGGGCGCGTGCCACGCCCGCGGGGTGAAGTCGGAGCGCGGGGCCTGCTGGAGAGAGGAGCGCTGCGGACCGAGGTGAGTGTGTGAGGCGCAGGCTGGGCCCTCCAGAGCCGCGGCTCGTCCTCGCCCACCTGCGTCCTGGCCGGTCCAGTGCTCAGCGGCAGTTGGGGCCTCCGCGCGCAGTGTGAAACCCGGACGTGGCAGGGCGTGTCCGCGCCCGACCGACCCTCAGCTGCTGGGGCGAAAACACAAGTGGACCTCAGTC

**Inserted sequence of E2 luciferase reporters**

GGTTATGAAATTCAATGCCCCCTTCCTCTGAACTCCTGCAAATCTCCAGTAAAGCACCACAGATTGACGAATATTCCAGCCATTTCCCTCTCCCGTACGCTAGCTCCATTTCCCACTTCTATCCATCTTCTCGAGCTTCCTCAGCTCCTCCACCTCCATGAGGCCTGGAAAGCACCTTGCTCCAGGAATGCGAGTAGGAGGCTCAGAGCGACCAGAAGTGCCAAAAGCTGACATTCAGATACTGCCGAAACAAACCACAAGAGCGCTAGGGCCCCCGCTAATAGTCCAGCTGCGATGGTGAGAACTCGCGGACCCGCCGGCGATGCATGCCGGGAGATGTAGTCCCGGGCCGACGCATTACCTCTTGGGAGTCGTGGTCTTCGATCTGGTAAACAGAAGACGGCAACTCGGTTAAGTCTTCCCCCACCGCCCCCTGAAATCCCTCGCCCCACCCGCGAGGGACTGGGGTAGGAGTAGGGGCGTTGCCGTGGTTAGCCTCGAACTCCTAGGCTCAGACGATACTCTCGCCTCGGCCTCCCGAGCAGCTGGGACTACACGCGTGAGCCACCGCCCCCGGCATAAAGTTTGAATTAGTCCTTACGCAAAAAGGGAAGAGGGCAGTCTGTAAACTCGCGCAGGATCAAGCTCTCGAGCTCCCGTCTTGGGTTAGCGCGCAGGGCGGAAGCGGGGAGAAGGCGGATCCGGGAGGCGGGGATACGTTACGTCGACGCGGGCGTGACCCTGGGCGAGAGGGTTTGGCGGGAATTCTGAAAGCCGCTGGCGGACCGCGCGCAGCGGCCAGAGACCGAGCCCTAAGGAGAGTGCGGCGCTTCCCGAGGCGTGCAGCTGGGAACTGCAACTCATCTGGGTTGTGCGCAGAAGGCTGGGGCAAGCGAGTAGAGAAGTGGAGCGTAAGCCAGGGGCGTTGGGGGCCGTGCGGGTCGGGCGCGTGCCACGCCCGCGGGGTGAAGTCGGAGCGCGGGGCCTGCTGGAGAGAGGAGCGCTGCGGACCGAGGTGAGTGTGTGAGGCGCAGGCTGGGCCCTCCAGAGCCGCGGCTCGTCCTCGCCCACCTGCGTCCTGGCCGGTCCAGTGCTCAGCGGCAGTTGGGGCCTCCGCGCGCAGTGTGAAACCCGGACGTGGCAGGGCGTGTCCGCGCCCGACCGACCCTCAGCTGCTGGGGCGAAAACACAAGTGGACCTCAGTCAAGCTT

**Inserted sequence of M1 luciferase reporters**

GCGTGATCTCGCTCACTGCAACCTCCACCTCCCGGGTTCAAGCACTTCTCTGCCTCAGCCTCCCAAAGAGCTGGGATTACAGGCATGCACCACCACGCCCGGCGTAGAGATGGGGTTTTGCCATCTTGGCCAGGCTGGTCTTGAACTCCTGACCTCGTGATCCGCCCACCTTGGCCTCCCAAAGTTCTGGGATTACAGGCTTGAGCCACCGCGCCTGGCCTCTCCTTACATGTTTTGTTGTTGTCGCTTGTCTTTTGAGACAAGGTCTCACTCTGTAGCCCAGGCTGGAGTGCAGTGGCCCATCATAGCTCACTGCAGCCTTGACGTCCTGAGCTCAAGCAGTCCTCCCACCTCAGCCTCCGGGATAGCTGAGACCACAGGCACAAGCCACCACGCCCAGCTTATTTTTTTTTTTTTTTTTTTTGAGATATGGGGTCCCACTATGTTGCCCAGGCTGGTCTCCAACTCCTGAGCTCAAGCGATCCTCCCCCACCTTGGCTTCCCAAAGTGCTGGGATTACAGGTGTGAGCCACCGTGCAGGCCTTATATGATCTTCATATCCTGAACTAAATGTAACCTTCCAGTTTCGGCACTTGCTCTGGCACTTTTCCTCCCTCGCCAGATAATACTAATCTTTAATCATGTAGTTCGTTTCCATGCCCATACTACCCTATTTGCTTATAATGTCTTCCACTTCGCCCAAGAATCCCTACTCAGCTAGCTTGTGGTGTTGTTTTGACACAGTCTCGCTCTGTCGCCCAGGCTGGAGTACAGCGGCGAGATCTCGGTTGGCTGCAACCTCCTCCTGAGTTCAAGCGATTCTCATGCCTCAGCCTTTCGAGTAGCTAGGATTACAGGCATGTGCCACAAAACCTGGCTAATTTTTGTATTTTTTACTAAAGACGAGGTTTCACCACGTTGGCCAGGTTTATCTCCAACCCCTGACCTCAGGTGATCCGCCTGCCTTGGCCTCCTAAACTGCTGGGATTACTGGCGTGAACCACCGCGCCCGGCCCTACTCAGCCTTTAAAACCGGAATCACGGGTCAAAACTTTCTGGTAAACCACGATACGGTTTAGGTTATGAAATTCAATGCCCCCTTCCTCTGAACTCCTGCAAATCTCCAGTAAAGCACCACAGATTGACGAATATTCCAGCCATTTCCCTCTCCCGTACGCTAGCTCCATTTCCCACTTCTATCCATCTTCTCGAGCTTCCTCAGCTCCTCCACCTCCATGAGGCCTGGAAAGCACCTTGCTCCAGGAATGCGAGTAGGAGGCTCAGAGCGACCAGAAGTGCCAAAAGCTGACATTCAGATACTGCCGAAACAAACCACAAGAGCGCTAGGGCCCCCGCTAATAGTCCAGCTGCGATGGTGAGAACTCGCGGACCCGCCGGCGATGCATGCCGGGAGATGTAGTCCCGGGCCGACGCATTACCTCTTGGGAGTCGTGGTCTTCGATCTGGTAAACAGAAGACGGCAACTCGGTTAAGTCTTCCCCCACCGCCCCCTGAAATCCCTCGCCCCACCCGCGAGGGACTGGGGTAGGAGTAGGGGCGTTGCCGTGGTTAGCCTCGAACTCCTAGGCTCAGACGATACTCTCGCCTCGGCCTCCCGAGCAGCTGGGACTACACGCGTGAGCCACCGCCCCCGGCATAAAGTTTGAATTAGTCCTTACGCAAAAAGGGAAGAGGGCAGTCTGTAAACTCGCGCAGGATCAAGCTCTCGAGCTCCCGTCTTGGGTTAGCGCGCAGGGCGGAAGCGGGGAGAAGGCGGATCCGGGAGGCGGGGATACGTTACGTCGACGCGGGCGTGACCCTGGGCGAGAGGGTTTGGCGGGAATTCTGAAAGCCGCTGGCGGACCGCGCGCAGCGGCCAGAGACCGAGCCCTAAGGAGAGTGCGGCGCTTCCCGAGGCGTGCAGCTGGGAACTGCAACTCATCTGGGTTGTGCGCAGAAGGCTGGGGCAAGCGAGTAGAGAAGTGGAGCGTAAGCCAGGGGCGTTGGGGGCCGTGCGGGTCGGGCGCGTGCCACGCCCGCGGGGTGAAGTCGGAGCGCGGGGCCTGCTGGAGAGAGGAGCGCTGCGGACCGAGGTGAGTGTGTGAGGCGCAGGCTGGGCCCTCCAGAGCCGCGGCTCGTCCTCGCCCACCTGCGTCCTGGCCGGTCCAGTGCTCAGCGGCAGTTGGGGCCTCCGCGCGCAGTGTGAAACCCGGACGTGGCAGGGCGTGTCCGCGCCCGACCGACCCTCAGCTGCTGGGGCGAAAACACAAGTGGACCTCAGTC

**Inserted sequence of M2 luciferase reporters**

GCGTGATCTCGCTCACTGCAACCTCCACCTCCCGGGTTCAAGCACTTCTCTGCCTCAGCCTCCCAAAGAGCTGGGATTACAGGCATGCACCACCACGCCCGGCTAATTTTTGTAGTTTTAGTAGAGATGGGGTTTTGCCATCTTGGCCAGGCTGGTCTTGAACTCCTGACCTCGTGATCCGCCCACCTTGGCCTCCCAAAGTTCTGGGATTACAGGCTTGAGCCACCGCGCCTGGCCTCTCCTTACATGTTTTGTTGTTGTCGCTTGTCTTTTGAGACAAGGTCTCACTCTGTAGCCCAGGCTGGAGTGCAGTGGCCCATCATAGCTCACTGCAGCCTTGACGTCCTGAGCTCAAGCAGTCCTCCCACCTCAGCCTCCGGGATAGCTGAGACCACAGGCACAAGCCACCACGCCCAGCTTATTTTTTTTTTTTTTTTTTTTGAGATATGGGGTCCCACTATGTTGCCCAGGCTGGTCTCCAACTCCTGAGCTCAAGCGATCCTCCCCCACCTTGGCTTCCCAAAGTGCTGGGATTACAGGTGTGAGCCACCGTGCAGGCCTTATATGATCTTCATATCCTGAACTAAATGTAACCTTCCAGTTTCGGCACTTGCTCTGGCACTTTTCCTCCCTCGCCAGATAATACTAATCTTTAATCATGTAGTTCGTTTCCATGCCCATACTACCCTATTTGCTTATAATGTCTTCCACTTCGCCCAAGAATCCCTACTCAGCTAGCTTGTGGTGTTGTTTTGACACAGTCTCGCTCTGTCGCCCAGGCTGGAGTACAGCGGCGAGATCTCGGTTGGCTGCAACCTCCTCCTGAGTTCAAGCGATTCTCATGCCTCAGCCTTTCGAGTAGCTAGGATTACAGGCATGTGCCACAAAACCTGGCACGAGGTTTCACCACGTTGGCCAGGTTTATCTCCAACCCCTGACCTCAGGTGATCCGCCTGCCTTGGCCTCCTAAACTGCTGGGATTACTGGCGTGAACCACCGCGCCCGGCCCTACTCAGCCTTTAAAACCGGAATCACGGGTCAAAACTTTCTGGTAAACCACGATACGGTTTAGGTTATGAAATTCAATGCCCCCTTCCTCTGAACTCCTGCAAATCTCCAGTAAAGCACCACAGATTGACGAATATTCCAGCCATTTCCCTCTCCCGTACGCTAGCTCCATTTCCCACTTCTATCCATCTTCTCGAGCTTCCTCAGCTCCTCCACCTCCATGAGGCCTGGAAAGCACCTTGCTCCAGGAATGCGAGTAGGAGGCTCAGAGCGACCAGAAGTGCCAAAAGCTGACATTCAGATACTGCCGAAACAAACCACAAGAGCGCTAGGGCCCCCGCTAATAGTCCAGCTGCGATGGTGAGAACTCGCGGACCCGCCGGCGATGCATGCCGGGAGATGTAGTCCCGGGCCGACGCATTACCTCTTGGGAGTCGTGGTCTTCGATCTGGTAAACAGAAGACGGCAACTCGGTTAAGTCTTCCCCCACCGCCCCCTGAAATCCCTCGCCCCACCCGCGAGGGACTGGGGTAGGAGTAGGGGCGTTGCCGTGGTTAGCCTCGAACTCCTAGGCTCAGACGATACTCTCGCCTCGGCCTCCCGAGCAGCTGGGACTACACGCGTGAGCCACCGCCCCCGGCATAAAGTTTGAATTAGTCCTTACGCAAAAAGGGAAGAGGGCAGTCTGTAAACTCGCGCAGGATCAAGCTCTCGAGCTCCCGTCTTGGGTTAGCGCGCAGGGCGGAAGCGGGGAGAAGGCGGATCCGGGAGGCGGGGATACGTTACGTCGACGCGGGCGTGACCCTGGGCGAGAGGGTTTGGCGGGAATTCTGAAAGCCGCTGGCGGACCGCGCGCAGCGGCCAGAGACCGAGCCCTAAGGAGAGTGCGGCGCTTCCCGAGGCGTGCAGCTGGGAACTGCAACTCATCTGGGTTGTGCGCAGAAGGCTGGGGCAAGCGAGTAGAGAAGTGGAGCGTAAGCCAGGGGCGTTGGGGGCCGTGCGGGTCGGGCGCGTGCCACGCCCGCGGGGTGAAGTCGGAGCGCGGGGCCTGCTGGAGAGAGGAGCGCTGCGGACCGAGGTGAGTGTGTGAGGCGCAGGCTGGGCCCTCCAGAGCCGCGGCTCGTCCTCGCCCACCTGCGTCCTGGCCGGTCCAGTGCTCAGCGGCAGTTGGGGCCTCCGCGCGCAGTGTGAAACCCGGACGTGGCAGGGCGTGTCCGCGCCCGACCGACCCTCAGCTGCTGGGGCGAAAACACAAGTGGACCTCAGTC

**Inserted sequence of M3 luciferase reporters**

GCGTGATCTCGCTCACTGCAACCTCCACCTCCCGGGTTCAAGCACTTCTCTGCCTCAGCCTCCCAAAGAGCTGGGATTACAGGCATGCACCACCACGCCCGGCGTAGAGATGGGGTTTTGCCATCTTGGCCAGGCTGGTCTTGAACTCCTGACCTCGTGATCCGCCCACCTTGGCCTCCCAAAGTTCTGGGATTACAGGCTTGAGCCACCGCGCCTGGCCTCTCCTTACATGTTTTGTTGTTGTCGCTTGTCTTTTGAGACAAGGTCTCACTCTGTAGCCCAGGCTGGAGTGCAGTGGCCCATCATAGCTCACTGCAGCCTTGACGTCCTGAGCTCAAGCAGTCCTCCCACCTCAGCCTCCGGGATAGCTGAGACCACAGGCACAAGCCACCACGCCCAGCTTATTTTTTTTTTTTTTTTTTTTGAGATATGGGGTCCCACTATGTTGCCCAGGCTGGTCTCCAACTCCTGAGCTCAAGCGATCCTCCCCCACCTTGGCTTCCCAAAGTGCTGGGATTACAGGTGTGAGCCACCGTGCAGGCCTTATATGATCTTCATATCCTGAACTAAATGTAACCTTCCAGTTTCGGCACTTGCTCTGGCACTTTTCCTCCCTCGCCAGATAATACTAATCTTTAATCATGTAGTTCGTTTCCATGCCCATACTACCCTATTTGCTTATAATGTCTTCCACTTCGCCCAAGAATCCCTACTCAGCTAGCTTGTGGTGTTGTTTTGACACAGTCTCGCTCTGTCGCCCAGGCTGGAGTACAGCGGCGAGATCTCGGTTGGCTGCAACCTCCTCCTGAGTTCAAGCGATTCTCATGCCTCAGCCTTTCGAGTAGCTAGGATTACAGGCATGTGCCACAAAACCTGGCACGAGGTTTCACCACGTTGGCCAGGTTTATCTCCAACCCCTGACCTCAGGTGATCCGCCTGCCTTGGCCTCCTAAACTGCTGGGATTACTGGCGTGAACCACCGCGCCCGGCCCTACTCAGCCTTTAAAACCGGAATCACGGGTCAAAACTTTCTGGTAAACCACGATACGGTTTAGGTTATGAAATTCAATGCCCCCTTCCTCTGAACTCCTGCAAATCTCCAGTAAAGCACCACAGATTGACGAATATTCCAGCCATTTCCCTCTCCCGTACGCTAGCTCCATTTCCCACTTCTATCCATCTTCTCGAGCTTCCTCAGCTCCTCCACCTCCATGAGGCCTGGAAAGCACCTTGCTCCAGGAATGCGAGTAGGAGGCTCAGAGCGACCAGAAGTGCCAAAAGCTGACATTCAGATACTGCCGAAACAAACCACAAGAGCGCTAGGGCCCCCGCTAATAGTCCAGCTGCGATGGTGAGAACTCGCGGACCCGCCGGCGATGCATGCCGGGAGATGTAGTCCCGGGCCGACGCATTACCTCTTGGGAGTCGTGGTCTTCGATCTGGTAAACAGAAGACGGCAACTCGGTTAAGTCTTCCCCCACCGCCCCCTGAAATCCCTCGCCCCACCCGCGAGGGACTGGGGTAGGAGTAGGGGCGTTGCCGTGGTTAGCCTCGAACTCCTAGGCTCAGACGATACTCTCGCCTCGGCCTCCCGAGCAGCTGGGACTACACGCGTGAGCCACCGCCCCCGGCATAAAGTTTGAATTAGTCCTTACGCAAAAAGGGAAGAGGGCAGTCTGTAAACTCGCGCAGGATCAAGCTCTCGAGCTCCCGTCTTGGGTTAGCGCGCAGGGCGGAAGCGGGGAGAAGGCGGATCCGGGAGGCGGGGATACGTTACGTCGACGCGGGCGTGACCCTGGGCGAGAGGGTTTGGCGGGAATTCTGAAAGCCGCTGGCGGACCGCGCGCAGCGGCCAGAGACCGAGCCCTAAGGAGAGTGCGGCGCTTCCCGAGGCGTGCAGCTGGGAACTGCAACTCATCTGGGTTGTGCGCAGAAGGCTGGGGCAAGCGAGTAGAGAAGTGGAGCGTAAGCCAGGGGCGTTGGGGGCCGTGCGGGTCGGGCGCGTGCCACGCCCGCGGGGTGAAGTCGGAGCGCGGGGCCTGCTGGAGAGAGGAGCGCTGCGGACCGAGGTGAGTGTGTGAGGCGCAGGCTGGGCCCTCCAGAGCCGCGGCTCGTCCTCGCCCACCTGCGTCCTGGCCGGTCCAGTGCTCAGCGGCAGTTGGGGCCTCCGCGCGCAGTGTGAAACCCGGACGTGGCAGGGCGTGTCCGCGCCCGACCGACCCTCAGCTGCTGGGGCGAAAACACAAGTGGACCTCAGTC

**Inserted sequence of RAD51 overexpression plasmid**

atggcaatgcagatgcagcttgaagcaaatgcagatacttcagtggaagaagaaagctttggcccacaacccatttcacggttagagcagtgtggcataaatgccaacgatgtgaagaaattggaagaagctggattccatactgtggaggctgttgcctatgcgccaaagaaggagctaataaatattaagggaattagtgaagccaaagctgataaaattctggctgaggcagctaaattagttccaatgggtttcaccactgcaactgaattccaccaaaggcggtcagagatcatacagattactactggctccaaagagcttgacaaactacttcaaggtggaattgagactggatctatcacagaaatgtttggagaattccgaactgggaagacccagatctgtcatacgctagctgtcacctgccagcttcccattgaccggggtggaggtgaaggaaaggccatgtacattgacactgagggtacctttaggccagaacggctgctggcagtggctgagaggtatggtctctctggcagtgatgtcctggataatgtagcatatgctcgagcgttcaacacagaccaccagacccagctcctttatcaagcatcagccatgatggtagaatctaggtatgcactgcttattgtagacagtgccaccgccctttacagaacagactactcgggtcgaggtgagctttcagccaggcagatgcacttggccaggtttctgcggatgcttctgcgactcgctgatgagtttggtgtagcagtggtaatcactaatcaggtggtagctcaagtggatggagcagcgatgtttgctgctgatcccaaaaaacctattggaggaaatatcatcgcccatgcatcaacaaccagattgtatctgaggaaaggaagaggggaaaccagaatctgcaaaatctacgactctccctgtcttcctgaagctgaagctatgttcgccattaatgcagatggagtgggagatgccaaagac
